# A ferrocene-containing nucleoside analogue targets DNA replication in pancreatic cancer cells

**DOI:** 10.1093/mtomcs/mfac041

**Published:** 2022-06-11

**Authors:** Marium Rana, Alessio Perotti, Lucy M Bisset, James D Smith, Emma Lamden, Zahra Khan, Media K Ismail, Katherine Ellis, Katie A Armstrong, Samantha L Hodder, Cosetta Bertoli, Leticia Meneguello, Robertus A M de Bruin, Joanna R Morris, Isolda Romero-Canelon, James H R Tucker, Nikolas J Hodges

**Affiliations:** School of Biosciences, The University of Birmingham, Edgbaston, Birmingham, B15 2TT, UK; School of Chemistry, The University of Birmingham, Edgbaston, Birmingham, B15 2TT, UK; School of Biosciences, The University of Birmingham, Edgbaston, Birmingham, B15 2TT, UK; School of Biosciences, The University of Birmingham, Edgbaston, Birmingham, B15 2TT, UK; School of Biosciences, The University of Birmingham, Edgbaston, Birmingham, B15 2TT, UK; School of Biosciences, The University of Birmingham, Edgbaston, Birmingham, B15 2TT, UK; School of Biosciences, The University of Birmingham, Edgbaston, Birmingham, B15 2TT, UK; Department of pharmacy, college of pharmacy, Knowledge University, 44001 Erbil, Kurdistan Region, Iraq; Institute of Cancer and Genomic Sciences, and The University of Birmingham, Edgbaston, Birmingham, B15 2TT, UK; School of Biosciences, The University of Birmingham, Edgbaston, Birmingham, B15 2TT, UK; School of Biosciences, The University of Birmingham, Edgbaston, Birmingham, B15 2TT, UK; MRC Laboratory or Molecular Cell Biology, University College London, London, WC1E 6BT, UK; MRC Laboratory or Molecular Cell Biology, University College London, London, WC1E 6BT, UK; MRC Laboratory or Molecular Cell Biology, University College London, London, WC1E 6BT, UK; Institute of Cancer and Genomic Sciences, and The University of Birmingham, Edgbaston, Birmingham, B15 2TT, UK; School of Pharmacy, The University of Birmingham, Edgbaston, Birmingham, B15 2TT, UK; School of Chemistry, The University of Birmingham, Edgbaston, Birmingham, B15 2TT, UK; School of Biosciences, The University of Birmingham, Edgbaston, Birmingham, B15 2TT, UK

**Keywords:** Ferrocene, DNA replication, nucleoside analogue, replication fork arrest, pancreatic cancer

## Abstract

Pancreatic ductal adenocarcinoma (PDAC) is a disease that remains refractory to existing treatments including the nucleoside analogue gemcitabine. In the current study we demonstrate that an organometallic nucleoside analogue, the ferronucleoside 1-(*S*,*R*_p_), is cytotoxic in a panel of PDAC cell lines including gemcitabine-resistant MIAPaCa2, with IC_50_ values comparable to cisplatin. Biochemical studies show that the mechanism of action is inhibition of DNA replication, S-phase cell cycle arrest and stalling of DNA-replication forks, which were directly observed at single molecule resolution by DNA-fibre fluorography. In agreement with this, transcriptional changes following treatment with 1-(*S*,*R*_p_) include activation of three of the four genes (*HUS1*, *RAD1*, *RAD17*) of the 9-1-1 check point complex clamp and two of the three genes (*MRE11*, *NBN*) that form the MRN complex as well as activation of multiple downstream targets. Furthermore, there was evidence of phosphorylation of checkpoint kinases 1 and 2 as well as RPA1 and gamma H2AX, all of which are considered biochemical markers of replication stress. Studies in p53-deficient cell lines showed activation of *CDKN1A* (p21) and *GADD45A* by 1-(*S*,R_p_) was at least partially independent of p53. In conclusion, because of its potency and activity in gemcitabine-resistant cells, 1-(*S*,*R*_p_) is a promising candidate molecule for development of new treatments for PDAC.

## Graphical Abstract

**Graphical Abstract:**
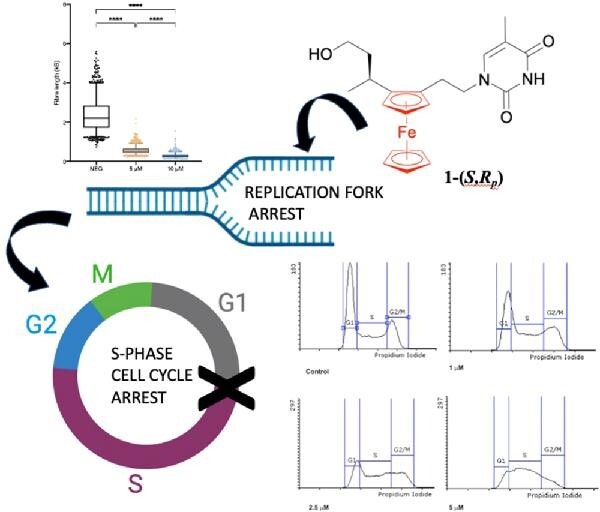
1-(*S*,*R*_p_) stalls DNA replication forks resulting in S-phase cell cycle arrest.

## Introduction

Pancreatic ductal adenocarcinoma (PDAC) is a disease with a very poor prognosis. Even when diagnosed early, the 5 year survival rate is less than 10%, the lowest of the 21 most common cancers in the UK.^1^ One important factor that contributes to this poor outlook is resistance to existing drugs and a lack of targeted therapies. Single-agent chemotherapies using nucleobase derivatives such as 5-fluorouracil and nucleoside analogues such as gemcitabine were first developed in the 1990s. Since then, progress has been made with the introduction of combination treatments such as FOLFOX and FOLFIRONOX^2^ and gemcitabine in combination with paclitaxel.^3^ Although these developments have resulted in modest but significant improvements in patient outcome,^4^^,^  ^5^ their clinical use is limited because of issues with toxic side effects. The outlook for patients with PDAC remains very poor and there is an urgent need for new compounds with activity in pancreatic cancer.

One strategy to overcome chemoresistance is to develop new chemical entities distinct from existing clinically used drugs with novel structural features and chemical composition.^6^ In this respect, metal complexes have proved an attractive starting point for the development of new drugs.^7^ One such group of compounds that have attracted considerable interest are those based on the organometallic compound ferrocene,^8^^-^^10^ which was shown to have anticancer activity as early as the 1980s.^11^ Since then, ferrocene-based compounds have been investigated for antihelminthic,^12^ antibacterial,^13^ antifungal,^14^ antimalarial^15^ and anticancer^16^ properties. As far as anticancer effects are concerned, perhaps the most successful example to date is that of ferrocifen, a ferrocene derivative combining the anti-estrogenic activity of tamoxifen with the cytotoxicity of ferrocene.^8^^,^^17^^,^^18^ Studies have shown that ferrocifen and its derivatives not only enhance the activity of tamoxifen in oestrogen receptor (ER) positive breast cancer cells, potentially limiting the emergence of tamoxifen resistance but are also toxic to ER receptor negative cells, suggesting ER-independent mechanisms of toxicity. Modification of naturally occurring nucleosides to generate analogues with antiviral and anticancer activities has also been a highly successful strategy in drug discovery as far as organic compounds are concerned.^19^ Our laboratory is developing new metal-containing nucleoside analogues containing ferrocene, termed ferronucleosides. In these compounds, the five-membered cyclopentadienyl ring of ferrocene acts as a bioisostere of the furanose ring in a nucleoside. We have developed compound 1-(*S*,*R*_p_), which contains both a thymine nucleobase and a hydroxyalkyl moiety linked to a ferrocene unit,^20^ making it chemically distinct from both 5-fluorouracil and gemcitabine. Previous detailed structure activity relationship (SAR) work in our group has shown that 1-(*S*,*R*_p_) is the most potent ferronucleoside identified. Interestingly, its ruthenocene analogue shows greatly reduced cytoxicity.^21^ However, other multiple studies have proven that cytotoxicity cannot be simply ascribed to the ferrocene moiety alone.^20^^,^^22^^,^^23^ For example, both the hydroxyalkyl linker and the thymidine base are required for effective cytotoxicity,^20^ which is also affected by the length of each linker.^22^ Furthermore, its regioisomer, in which both Cp rings are functionalized instead of one being doubly substituted, is significantly less cytotoxic.^23^

Our extensive SAR studies have given pointers as to the mode of action of 1-(*S*,*R*_p_). Work using non-phosphorylatable analogues and thymidine kinase–deficient cell lines^23^ suggests that although inhibition of DNA replication is involved, substrate phosphorylation is not required for toxicity, unlike classic nucleoside analogues such as gemcitabine. Therefore in order to probe this new mode of action further, while at the same exploring potential therapeutic applications towards cancers of unmet need, here we show that 1-(*S*,*R*_p_) has cytotoxic activity in multiple PDAC cell lines, including gemcitabine-resistant MIAPaCa2 cells. Moreover we report, for the first time, detailed mechanistic, biochemical and gene-expression studies on 1-(*S*,*R*_p_), which identify that its mode of action is distinct from gemcitabine, involving inhibition of DNA replication without incorporation into DNA. We also show that 1-(*S*,*R*_p_) causes induction of single-stranded DNA breaks in newly synthesized DNA, resulting in replication fork stress, S-phase arrest and apoptosis. We conclude that because of its potency, activity in gemcitabine-resistant cells and novel mode of action, 1-(*S*,*R*_p_) is a promising candidate molecule for development of new treatments for PDAC.

## Methods

### Cell culture

MIAPaCa2 (85062806), BxPC3 (93120816), CFPAC-1 (91112501), PANC-1 (87092802) pancreatic ductal adenoma cancer cell lines and HCT 116 p53 wild colorectal cells (91091005) were all purchased from the European Collection of Authenticated Cell Cultures. HCT 116 p53 null cells were generated as described previously^24^ and were a gift from Peter Sadler (University of Warwick). All of the PDAC cell lines studied contain verified homozygous missense substitution mutations in the DNA binding domain of TP53 as catalogued in the COSMIC database of cancer cell lines (https://cancer.sanger.ac.uk/cell_lines) as follows: MIAPaCa2 (pR248W, c742 C→T), BxPC3 (pY220C, c659 A→G), CFPAC-1 (pC242R, c724 T→C) and PANC-1 (pR273H, c818 G→A). All of these mutations are classified as non-functional in the IARC TP53 database (http://p53.iarc.fr) based on overall transcriptional activity using eight different promoters measured in yeast^25^ The presence of published TP53 mutations was confirmed directly by sequencing (Supplementary Information). All cell culture media and supplements were purchased from Gibco (Thermo Scientific, Waltham, MA, USA) and all plasticware was purchased from Greiner Bio-One. All lines were grown in T_75_ tissue culture flasks as monolayers. MIAPaCa2 cells were cultured in DMEM, supplemented with 10% (v/v) foetal bovine serum, 100 U/mL penicillin, 100 mg/mL streptomycin, and 2 mM L-glutamine; BxPC3, PANC-1 and CFPAC-1 were cultured in RPMI-1640 and supplemented in the same way. All cells were maintained at 37°C in a 5% CO_2_ humidified incubator and sub-cultured twice weekly before confluency. All cell cultures were confirmed free from *Mycoplasma sp*. contamination using the EZ-PCR mycoplasma detection kit according to the manufacturer's instructions. All cells were cultured up to passage 20 before being discarded.

### MTT assay

Compound 1-(*S*,*R*_p_) was prepared as 50 mM stock solutions in dimethylsulfoxide (DMSO), cisplatin and gemcitabine HCl stocks were prepared in physiological saline. On the day of the experiment fresh solutions at the appropriate test concentration were prepared in cell culture media. For 1-(*S*,*R*_p_) the final concentration of DMSO was 0.5% v/v, which was also used as a vehicle control. The MTT [3-(4,5-dimethylthiazol-2-yl)-2,5-diphenyltetrazolium bromide] assay was used to assess cell cytotoxicity.^26^ Cells were seeded at a density of 5000 cells per well in a 96-well plate, exposed to different concentrations of 1-(*S*,*R*_p_) and incubated for 72 h. MTT was added to a final concentration of 0.5 mg/mL followed by a 3 h incubation. DMSO was added to solubilize formazan and absorbance was read at 490 nm using Tecan Infinite F200 Pro platereader (Tecan, Männedorf, Switzerland). Samples were analysed in triplicate technical replicate and each experiment was repeated three times independently and data were normalized to the vehicle (DMSO) control.

### Gene-expression profiling

Total RNA was extracted from cells using a Qiagen RNeasy Mini Kit and reverse-transcribed using a Qiagen RT² First Strand Kit according to manufacturer's instructions. Gene expression of 84 key genes related to cell cycle progression and DNA-damage checkpoint were quantified using a Qiagen RT² Profiler™ PCR Array Human Cell Cycle PCR array (PAHS-020Z) according to manufacturer's instructions. For MIAPaCa2 cells, the PCR array was carried out in three independent biological replicates. For BxPC3 and CFPAC-1 cells, one biological replicate was undertaken. Results were analysed using the delta delta Cq method with normalization to *GAPDH*, which was the most stable housekeeping gene across samples. Thirteen genes (*BRCA2*, *HUS1*,*RBBP8*, *SERTAD1*, *MDM2*, *GADD45A*, *CASP3*, *CDKN1A*, *CDK2*, *CCNG2*, *CCNE1*, *CDK7* and CCNT1) were selected for further validation in all three cells by qPCR. Briefly, brilliant II SYBR® Green QPCR Master Mix (Agilent, Santa Clara, CA, USA) and using the cycling conditions in the kit recommended for Agilent Aria MX. Primers used for each gene of interest are shown in the supplementary materials and methods. B2M was the housekeeping gene of choice used as an internal control.

### Comet assay

After treatment, cells were trypsinized and centrifuged (5 min, 800× *g*) to remove the culture medium. Cells were then resuspended in 90 μL low melting agarose 0.7% (LMA) and quickly layered onto degreased microscope slides previously dipped in 1% normal melting agarose for the first layer. The agarose was allowed to set for 15 min at 4°C before the addition of a final 90 μL layer of LMA. Cell lysis was carried out at 4°C overnight by incubating the slides in lysis buffer (2.5 M NaCl, 100 mM Na2EDTA, 8 mM Tris-HCl, 1% Triton X-100 and 10% DMSO, pH  =  10). At the end of the lysis period, slides were placed horizontally in a block electrophoretic chamber and quickly submerged with electrophoretic alkaline buffer, pH > 13 (1 mM Na_2_EDTA, 300 mM NaOH, 0°C) and incubated for 20 min at 4°C to allow unwinding of DNA. The electrophoretic migration was performed for 20 min at 0.78 V cm^−1^ and 300 mA. After electrophoresis, each slide was neutralized with 2 mL of neutralization buffer (0.4 M Tris-HCl, pH 7.5), fixed in ethanol at −20°C and left to dry for at least 4 h. DNA was stained with 75 μL SYBR™ Gold Nucleic Acid Gel Stain before the examination at 200× magnification under a Leica DMLS fluorescence microscope (Leica Microsystems GmbH, Wetzlar, Germany; excitation filter BP 515–560 nm, barrier filter LP 580 nm), using an automatic image analysis system (Comet Assay IV—Perceptive Instruments Ltd., Stone, Staffordshire, UK). In order to assess if the location of DNA-strand breaks caused by 1-(*S*,*R*_p_) treatment coincided with 5-Ethynyl-2'-deoxyuridine (EdU) incorporation sites after treatment, cells were pulse-labelled with EdU 10 μM before been harvested. Slides for the comet assay were prepared as usual and then labelled with AlexaFluor488 picolyl azide after the final dehydration prior to staining with SYBR Gold.

### CellTrace analysis

Cellular proliferation was quantified by quantifying the time-dependent decrease in CellTrace Far Red fluorescence labelling as the result of cell division using a CellTrace cell proliferation kit (Thermo Fisher Scientific, USA, cat. no. C34564) according to manufacturer's instructions. Briefly, cells were pulse labelled with CellTrace Far Red (10 μM, 30 min) before treatment with 1-(*S*,*R*_p_) (0–5 μM) for 72 h. At the end of the experiment, cells were suspended by trypsinization and fluorescence analysed by flow cytometry (BD FACSCalibur, BD Biosciences, USA).

### EdU labelling

Nucleotide incorporation rate was assessed through the Click-iT™ Plus EdU Alexa Fluor™ 488 Flow Cytometry Assay Kit (Thermo Scientific) according to manufacturer's specification. Briefly, EdU incorporation was detected through click labelling with Alexafluor488 picolyl azide and measured through both a BD FACSCalibur™ flow cytometer (Becton Dickinson, Franklin Lakes, NJ, USA) equipped with a 488 nm argon laser and a 635 nm red diode laser and through a Nikon A1R Inverted Confocal/TIRF microscope (Nikon, Minato, Tokyo, Japan). DNA content in the cell population was counterstained with eBioscience™ 7-AAD Viability Staining Solution (Thermo Scientific) according to manufacturer's specifications. Single dye-stained samples were run prior to the analysis to ensure no signal compensation was necessary. Flow cytometry samples were analysed in triplicate technical replicate and every experiment was repeated three times.

### Cell cycle analysis

After treatment with different concentrations of 1-(*S*,*R*_p_), cells were harvested, fixed and permeabilized as described previously.^27^ After centrifugation, cells were resuspended in phosphate buffered saline (PBS) followed by incubation with propidium iodide (0.01 mg/mL) and RNAse A (1 mg/mL) (Qiagen, Germany; cat. no. 19101) for 30 min. The samples were analysed by flow cytometry using BD FACSCalibur (BD Biosciences) in triplicate technical replicate and every experiment was repeated three times.

### DNA-fibre fluorography analysis

MIAPaCa2 cells were seeded at a density of 3 × 10^5^ cells/mL and incubated in 5% CO_2_, 37°C overnight. Cells were treated with 1-(*S*,*R*_p_) for 24 h. Following treatment, cells were pulse labelled with 25 μM CldU for 20 min followed by second labelling with 250 μM IdU for an additional 20 min. Cells were harvested and the fibres were spread as described previously.^28^ DNA fibres were immunolabelled to detect IdU with mouse anti-BrdU (1:70; Becton Dickinson) and CldU with rat anti-BrdU (1:200; Abcam, UK) antibodies and incubated overnight at 4°C. Following fixation in 4% paraformaldehyde, the slides were incubated with secondary antibodies goat anti-rat Alexafluor 555 (1:500; ThermoFisher Scientific) and goat anti-mouse Alexafluor 488 (1:500; ThermoFisher Scientific) for 90 min. The images were captured using confocal microscopy with an oil immersion objective and analysed using ImageJ. At least 1000 fibres per condition from three independent experiments were quantified by measuring the combined length (micrometre) of the red- and green-fluorescent label. Fibre length in micrometres was then expressed as kB of DNA as described previously^29^ where 1 μm is estimated to be 2.59 kB of extended DNA.

### Gamma-H2AX staining

MIA PaCa-2 cells were treated with 10 μM 1-(*S*,*R*_p_) or positive control etoposide 5 μM for 24 h for comparison. Negative controls were treated with 0.5% DMSO (Sigma, Ireland), vehicle solvent. After treatment, cells were detached using standard trypsin/EDTA protocol and cell pellets were obtained. Cells were fixed in ice-cold 70% ethanol and stored overnight at 4°C. Fixed cells were centrifuged and resuspended in 0.25% Triton X-100 (Sigma; cat. no. T8787) for 10 min. After centrifugation, cells were resuspended in blocking solution (2% bovine serum albumin (BSA)) for 30 min at room temperature (RT). Blocking solution was removed by centrifugation and exposed to the anti-gamma H2AX (phospho S139) antibody (1:500) (Abcam; cat. no. ab26350) in blocking solution for 1 h at RT. The primary antibody was removed, cells were washed and incubated with the goat anti-mouse fuorescein isothiocyanate (FITC) antibody (1:200) (ThermoFisher Scientific; cat. no. 62-6511) in blocking solution for 1 h in the dark at RT. After removing the secondary antibody and washing, the cell pellets were resuspended in 1 mL blocking solution. The samples were analysed by flow cytometry using BD FACSCalibur (BD Biosciences) in triplicate technical replicate and every experiment was repeated three times.

For confocal analysis, cells were fixed in 4% paraformaldehyde on a rocker for 10 min at RT followed by incubation with 0.1% Triton X-100 (Sigma) for 10 min. After washing with phosphate buffered saline 0.1% v/v Tween 20 (PBST), cells were blocked with 2% BSA in PBST for 1 h at RT. Cells were washed and incubated with anti-gamma H2AX (phospho S139) antibody (1:500) (Abcam; cat. no. ab26350) overnight at 4°C in a humidified chamber. Cells were washed and incubated with goat anti-mouse (FITC) antibody (1:200) (ThermoFisher Scientific) (cat. no. 62-6511) for 1 h in the dark at RT. After PBST wash, cells were incubated with propidium iodide (0.01 mg/mL) for 10 min. Samples were washed again and coverslips (ThermoFisher Scientific) were applied using hydromount mounting media. The slides were allowed to dry for 1 h in the dark at RT. The slides were read using Nikon A1R Inverted Confocal/TIRF microscope (Nikon) using a ×100 oil-immersion objective.

## Results and discussion

### 1-(*S*,*R*_p_) cytotoxicity in PDAC cell lines is comparable to cisplatin

**Fig. 1:**
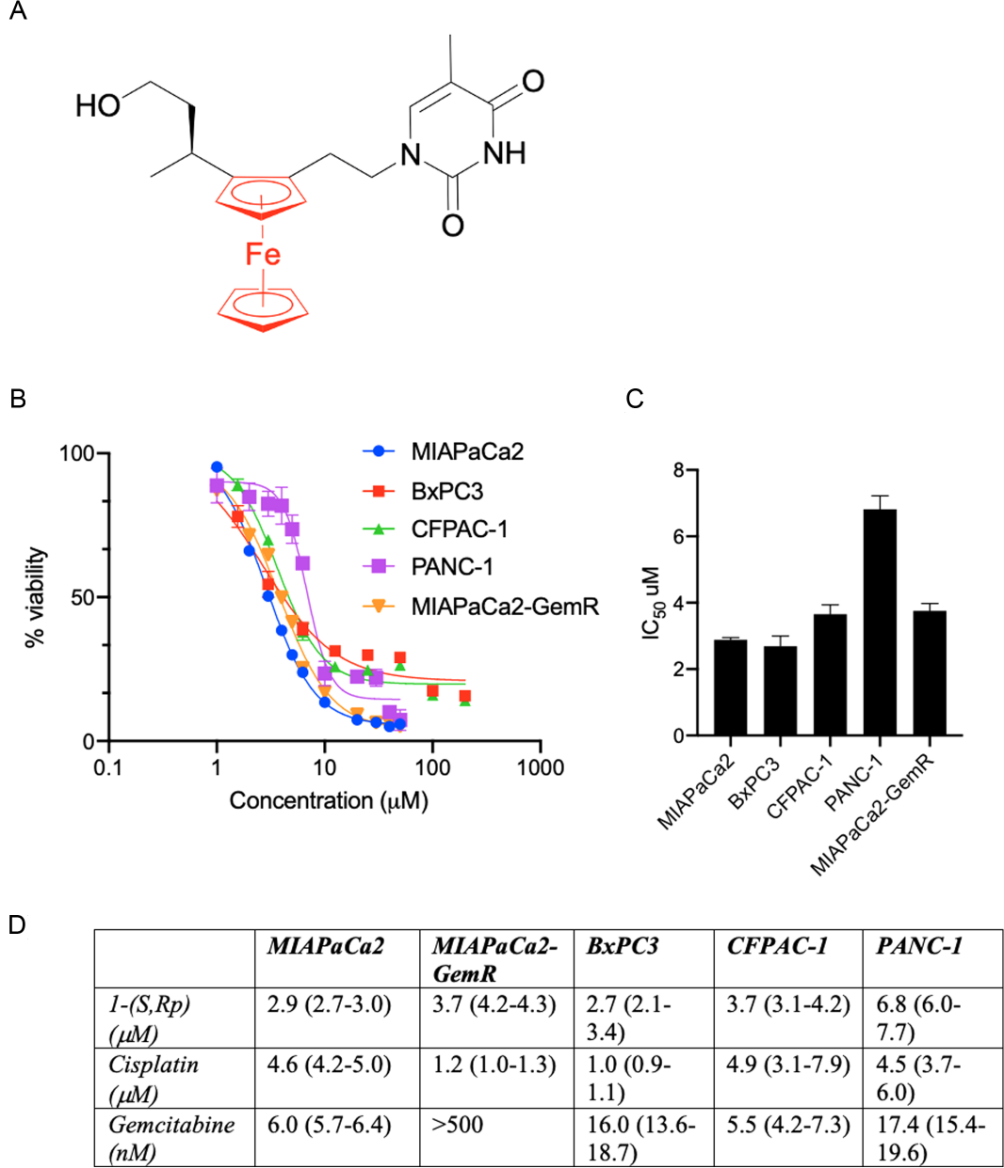
Compound 1*-*(*S*,*R*_p_) whose chemical structure is shown in (A) with ferrocene highlighted in red is cytotoxic to a panel of pancreatic ductal adenoma carcinoma cells. (B) Cytotoxicity curves in pancreatic ductal adenoma carcinoma (PDAC) cell lines treated with 1-(*S*,*R*_p_) for 72 h as assessed by the MTT [3-(4,5-dimethylthiazol-2-yl)-2,5-diphenyltetrazolium bromide] assay. The results represent the mean of three independent biological experiments (*n* = 3). (C) Modelled IC_50_ (±SD) values of 1-(*S*,*R*_p_) (variable slope 4 parameter). (D) Comparison of 72 h IC_50_ values for 1-(*S*,*R*_p_), cisplatin expressed in micromoles and gemcitabine HCl, expressed in nanomoles in the panel of five pancreatic ductal adenoma cells. The values in parentheses are the 95% CIs. All results represent the mean from three independent biological experiments (*n* = 3) and are normalized to vehicle control.

The nucleoside analogue (*S*,*R*_p_)-1-[α-methyl-(3-(hydroxy)propyl)]-2-[(thyminyl)ethyl]-ferrocene, herein referred to as 1-(*S*,*R*_p_) (Fig. 1A) was synthesized as described previously.^20^ Chiral purity was confirmed using high performance liquid chromatography (Supplementary Fig. S1). Previously, we reported that 1-(*S*,*R*_p_) is cytotoxic to multiple cancer cell lines including those from the GI tract with a potency comparable to, or superior to cisplatin.^20^ We also demonstrated that non-phosphorylatable analogues of 1-(*S*,*R*_p_) have similar cytotoxicity to the parent compound,^23^ indicating a novel mode of action for a nucleoside analogue that does not proceed via substrate phosphorylation and incorporation into genomic DNA. Here, we conducted detailed studies to further probe the mode of action of 1-(*S*,*R*_p_), while at the same time assessing for activity in PDAC, a cancer of unmet need. Cytotoxicity of 1-(*S*,*R*_p_) in five PDAC cell lines was compared to gemcitabine, the frontline treatment for PDAC since its approval by the FDA in 1996^30^ and cisplatin, a reference metallo-drug used to treat a range of solid neoplasms.^31^ Compound 1-(*S*,*R*_p_) was cytotoxic to all five PDAC cell lines investigated, with calculated 72 h IC_50_ values of 2.9, 3.7, 2.7, 3.7 and 6.8 μM in MIAPaCa2, MIAPaCa2-GemR, BxPC3, CFPAC-1 and PANC-1 cell lines, respectively (Fig. 1B and C). In contrast, we have shown previously that 1-(*S*,*R*_p_) is not toxic to normal human embryonic lung cells.^20^ In future studies, it would be useful to compare activity to normal pancreatic cells as well. An important finding was that there was no statistically significant difference between the IC_50_ value of 1-(*S*,*R*_p_) in MiaPaCa2 and gemcitabine-resistant MIAPaCa2 cells (Fig. 1D). The potency of 1-(*S*,*R*_p_) was lower than that of gemcitabine, which had 72 h IC_50_ values in the nanomole range except in gemcitabine-resistant MIAPaCa2 cells (Fig. 1D). In all cell lines tested, the IC_50_ value of 1-(*S*,*R*_p_) was comparable with or superior to cisplatin, which were 4.6, 1.2, 1.0, 4.9 and 4.5 μM in MiaPaCa2, MIAPaCa2-GemR, BxPC3, CFPAC-1 and PANC-1 cell lines, respectively (Fig. 1D). Analysis by flow cytometry confirmed that the mechanism of cell death is apoptosis (Supplementary Fig. S2).

### 1-(*S*,*R*_p_) arrests cells in S-phase and inhibits DNA replication

**Fig. 2:**
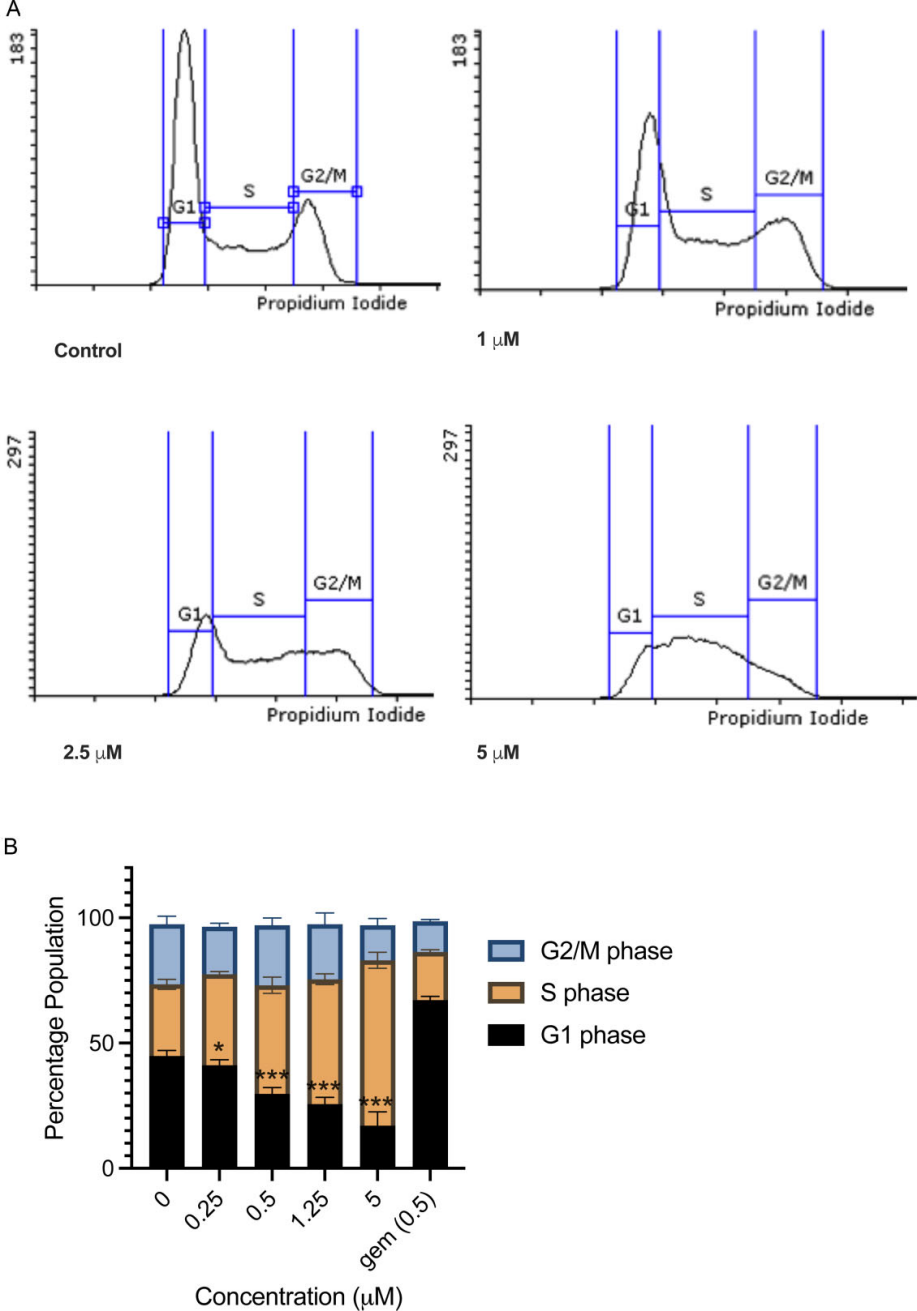
Compound 1-(*S*,*R*_p_) causes concentration-dependent S-phase arrest in MIAPaCa2 pancreatic ductal adenoma carcinoma cells. Cells were treated with 1-(*S*,*R*_p_) (0–5 μM) for 24 h before staining with propidium iodide and analysis by flow cytometry. (A) Representative histograms from cells treated with 0, 1, 2.5 and 5 μM 1-(*S*,*R*_p_). (B) Graphical representation of data, the results represent the mean of three independent biological experiments (±SD, *n* = 3). *, ** and *** statistically significantly different as assessed by 2-way ANOVA followed by Dunnett's multiple comparison *t*-test. As a positive control, cells were also treated with 0.5 μM gemcitabine.

Next, we investigated the effect of 1-(*S*,*R*_p_) on cell cycle progression and DNA replication. Treatment of MIAPaCa2 cells (0–5 μM, 24 h) resulted in accumulation in the S-phase of the cell cycle (Fig. 2A). The percentage of cells in S-phase were 29 ± 2.0%, 36 ± 1.2%, 43 ± 3.3%, 50.0 ± 2.2% and 66.0 ± 3.2% following treatment with 0, 0.25, 0.5, 1.25 and 5 μM 1-(*S*,*R*_p_), respectively (Fig. 2B). This effect was concentration dependent and statistically significant (*P* < 0.001, 2-way ANOVA followed by Dunnett's multiple comparison testing). As a positive control, cells were treated with gemcitabine (0.5 μM, 24 h), which resulted in an accumulation of 67.0 ± 1.5% cells in G1 phase of the cell cycle (Fig. 2B). Therefore, like 5-fluorouracil^32^ and gemcitabine,^33^ 1-(*S*,*R*_p_) has a profound effect on progress of cancer cells through the cell cycle.

**Fig. 3:**
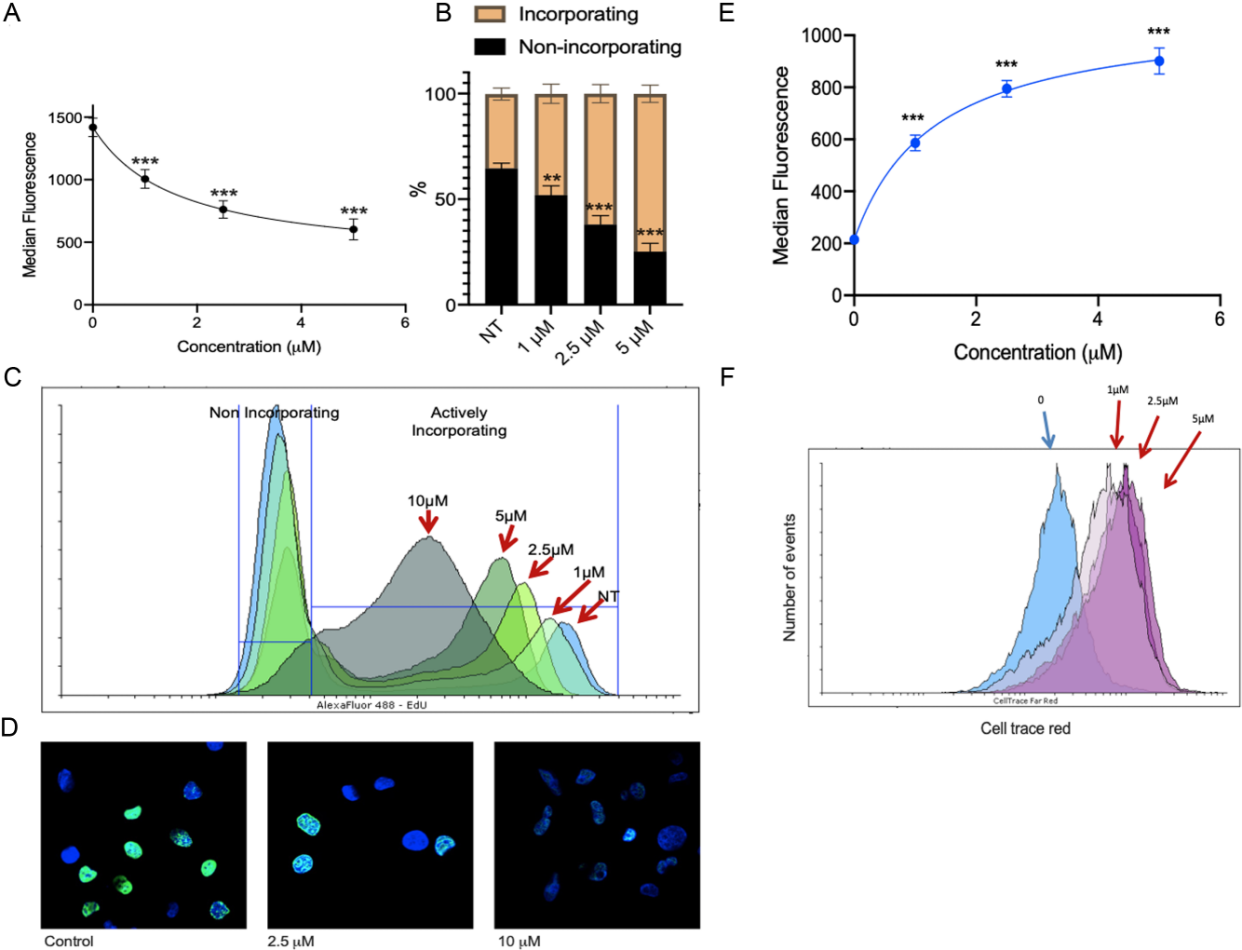
Compound 1-(*S*,*R*_p_) inhibits replication of genomic DNA in MIAPaCa2 pancreatic ductal adenoma carcinoma cells. (A) Incorporation of EdU into replicating DNA as assessed by flow cytometry following treatment of cells with 1-(*S*,*R*_p_) for 24 h. *** Significantly different from untreated control (1-way ANOVA followed by a *post hoc* Dunnett's *t*-test). (B) Percentage of EdU incorporating and non-incorporating cells following treatment with 1-(*S*,*R*_p_) (24 h) as quantified using the criteria outlined in (C) for a representative experiment. (D) Confocal microscopy confirms reduced EdU incorporation (green fluorescence) into genomic DNA in the nuclei of cells counter stained with DAPI (blue fluorescence). (E) Median cellular labelling with CellTrace Far Red. (F) Representative histograms from an individual experiment. The results represent the mean of three independent biological experiments (±SD, *n* = 3). *** Statistically significant from control (*P* < 0.001, 1-way ANOVA followed by a *post hoc* Dunnett's *t*-test). The calculated EC_50_ of 1-(*S*,*R*_p_) was 1.3 μM (95% CI 0.9–1.9 μM).

To gain further insight into the mechanism of accumulation of cells in S-phase, DNA replication and cell division were quantified. Following treatment of MIAPaCa2 cells with 1-(*S*,*R*_p_) there was a statistically significant concentration-dependent decrease in the median EdU fluorescence labelling of cells (*P* *<* 0.001, 1-way ANOVA) following treatment of cells with 1-(*S*,*R*_p_) (Fig. 3A). Median fluorescence values were 1418 ± 73, 1005 ± 74, 762 ± 69 and 603 ± 84 following treatment with 0, 1, 2.5 and 5 μM 1-(*S*,*R*_p_), respectively. Interestingly, although median EdU labelling decreased following treatment with 1-(*S*,*R*_p_), the number of cells actively incorporating EdU significantly increased in a concentration-dependent manner (*P* < 0.001, 2-way ANOVA followed by Dunnett's multiple comparison testing, Fig. 3B and C). Together, these data suggest that following treatment of cells with 1-(*S*,*R*_p_), cells accumulate in S-phase due to inefficient replication of genomic DNA and an increased residence in this phase of the cell cycle. This was further confirmed by confocal microscopy analysis, where it was observed that control cells were either strongly labelled with EdU or almost completely unlabelled. In contrast, in cells treated with 1-(*S*,*R*_p_), the overall level of EdU labelling in individual cells was reduced but a higher proportion of cells were labelled (Fig. 3D) confirming that cells spend longer in S-phase.

To further confirm that 1-(*S*,*R*_p_) inhibits cell and genomic DNA replication, cells were pulse labelled with CellTrace Far Red prior to treatment with 1-(*S*,*R*_p_) (0–5 μM) for 72 h. As shown in Fig. 3E and F, there was a statistically significant increase (*P* < 0.001) in intensity of labelling of cells with CellTrace Far Red compared to controls in a concentration-dependent manner, with a calculated EC_50_ of 1.3 μM (95% CI 0.9–1.9 μM) confirming that 1-(*S*,*R*_p_) inhibits cellular division, preventing dilution of the CellTrace label into newly replicated cells. Together these data suggest that the activity of 1-(*S*,*R*_p_) involves a specific interaction with a cellular target related to DNA replication. Further evidence that interaction with genomic DNA is a major mode of action was apparent from COMPARE analysis of the one-dose data obtained in the NCI60 panel of cancer cell lines. Out of the 29 compounds identified as having a similar (Pearson's correlation >0.5) profile to 1-(*S*,*R*_p_), 13 (45%) were compounds whose mechanisms of action was alkylation of guanine, 7 (24%) were compounds identified as inhibiting DNA synthesis and 3 (10%) were topoisomerase inhibitors. Interestingly, four out of the top six correlations (4 out of 6 rounds to 67%) had inhibition of DNA synthesis as the identified mode of action Supplementary Table S1.

### Transcriptomic profiling identifies key changes in DNA-damage response

**Fig. 4:**
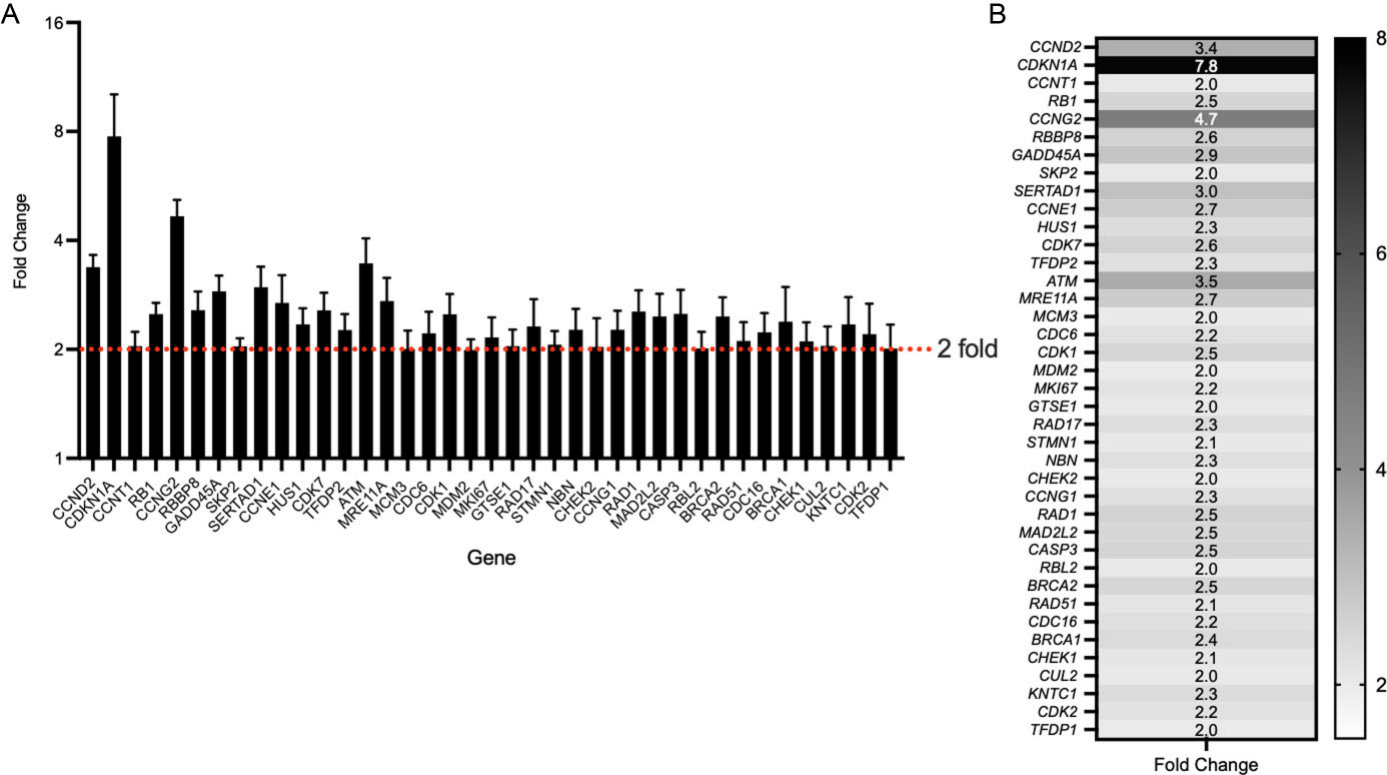
List of 39 genes related to DNA repair that are statistically significantly upregulated in MIAPaCa2 cells following treatment with 1-(*S*,*R*_p_) (10 μM, 24 h). (A) Graphed as fold change (2^−ddCq^) and (B) as a heat map for clarity. The results represent the mean of three independent biological experiments (±SD, *n* = 3). Statistically significantly changed genes plotted relative to *GAPDH* as 2^−dCq^ are shown in Supplementary Fig. S3.

To further study the mechanism of action, we quantified transcripts involved in cellular DNA-damage response. Of the 84 genes investigated 53 (63%) were upregulated more than 2-fold in MIAPaCa2 cells treated with 1-(*S*,*R*_p_) (10 μM, 24 h). Of these, 39 genes were statistically significantly upregulated (*P* < 0.05), confirming a strong transcriptional response in genes related to DNA-damage response. The complete list of upregulated genes is shown in Supplementary Table S2. Statistically significantly changed genes are plotted as fold change normalized to *GAPDH* (Fig. 4) and relative to *GAPDH* as 2^−dCq^ (Supplementary Fig. S3). Next, the same set of 84 genes were analysed in CFPAC-1 and BxPC3. overall, fewer changes in gene expression more than 2-fold were observed in six (7%) and nine (11%) instances, respectively. However, out of the top 12 most significantly changed genes observed in MIAPaCa2 cells, 9 (75%) genes were also upregulated in either BxPC3, CFPAC-1 or both cell lines (Supplementary Table S2). Furthermore, out of the 39 genes significantly upregulated in MIAPaCa2 cells 13 (33%) were also induced by at least 1.5-fold in at least one of the other two cell lines (Supplementary Table S2).

K-means clustering by String network analysis^34^ showed that the 39 genes upregulated in MIAPaCa2 cells clustered into two distinct groups (Supplementary Fig. S4). The first group contained transcripts related to DNA-double-strand break repair (Reactome pathway HSA-5693532) and DNA repair (Reactome pathway HSA-73894) and included the following genes *RAD17*,*RAD1*, *HUS1*, *NBN*, *MRE11A*, *CHEK1*, *CHEK2*,*RBBP8*, *ATM*, *BRCA1* and *MAD2L2*. The second cluster contained transcripts related to G1/S transition (Reactome pathway HSA-69206), S-phase (Reactome pathway HSA-69242) and mitotic G1-G1/S phase (Reactome pathway HSA-453279) and contained the genes *CDK1*, *CDK7*, *MCM3*, *CDC6*, *CDC16*, *CCNE1*, *CCND2*, *TFDP1*, *DP2*, *SKP2*,*RB1*, *CDK2*,*RBL2* and *CDKN1A.* Functional annotation analysis of significantly upregulated genes in DAVID revealed statistically significant (Benjamini corrected *P*-values <0.0001) over-representation of genes from the following BIOCARTA pathways: ATM signalling pathway (11/21 genes), role of BRCA1, BRCA2 and ATR in cancer susceptibility (11/16), cell cycle (8/25), cyclins and cell cycle regulation (8/25), p53 signalling pathway (7/17) and cell cycle (8/30). Together these data suggest a conserved transcriptional response to 1-(*S*,*R*_p_) in PDAC cells that is related to DNA-damage response and S-phase arrest.

**Fig. 5:**
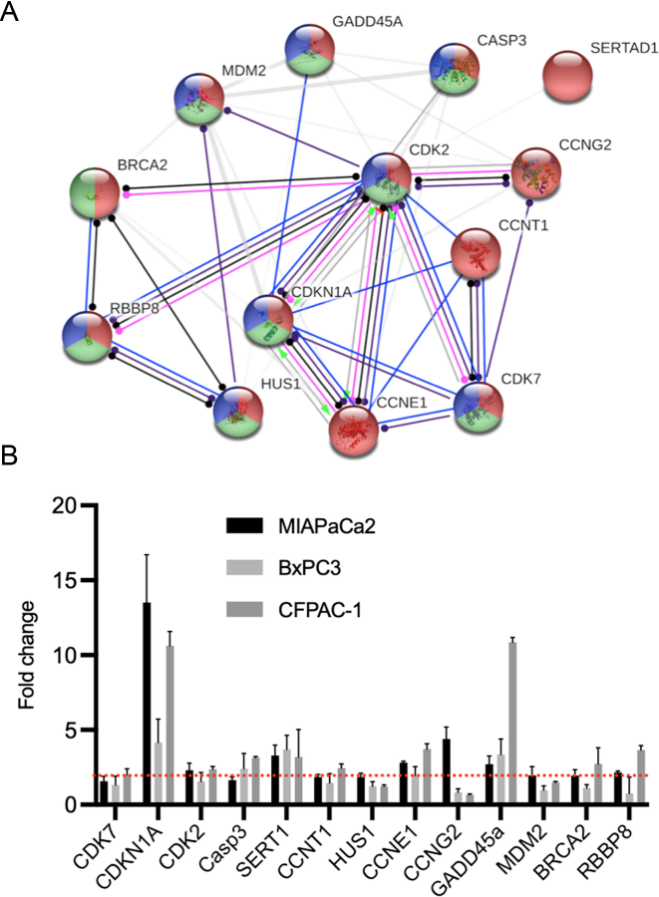
Compound 1-(*S*,*R*_p_) induces a conserved transcriptional response in three pancreatic ductal adenocarcinoma (PDAC) cell lines. (A) STRING network analysis (https://string-db.org) of the 13-genes transcriptional activated in MIAPaCa2 and at least one other PDAC cell line following treatment Within the network major GO erms represented included: GO:0051726 regulation of cell cycle (coloured red), GO:0045786 negative regulation of cell cycle (coloured blue) and GO:0006974 cellular response to DNA-damage stimulus (coloured green). Interconnecting lines within the network represent predicted molecular actions
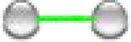
 activation , 
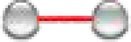
 inhibition, 
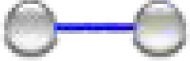
 binding, 
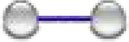
catalysis, 
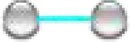
phenotype, 
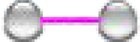
posttranslational modification, 
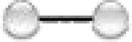
reaction and 
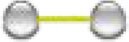
transcriptional regulation. (B) Quantification of fold changes as assessed by qPCR in MIAPaCa2, BxPC3 and CFPAC-1 cells following treatment with 1-(*S*,*R*_p_) (10 μM, 24 h). The red dotted line represents 2-fold increase compared to untreated control. The results represent the mean of three independent biological experiments (±SD, *n* = 3).

To gain further insight into the core transcriptional response we analysed the 13 significantly upregulated genes in MIAPaCa2 cells that were also upregulated in at least one of the other two cell lines investigated. Analysis of these genes showed strong evidence of molecular interactions. Within the 13 nodes of the network, the number of edges was 22 and the average local clustering coefficient was 0.588 and had a protein protein enrichment (PPI) enrichment value <1.0e-16. As shown in Fig. 5A, within the network the major gene ontology (GO) terms represented included: GO:0051726 regulation of cell cycle (coloured red), GO:0045786 negative regulation of cell cycle (coloured blue) and GO:0006974 cellular response to DNA-damage stimulus (coloured green). The top 10 significantly functionally enriched GO terms in the network are shown in Table 1. Together, these data strongly suggest that the underlying mechanism of response to 1-(*S*,*R*_p_) is the same in all three PDAC cell lines investigated and related to DNA-replication fork stress, DNA-damage response and cell cycle perturbation. To further validate these changes in gene expression, we quantified them by qPCR (Fig. 5B).

**Table 1.** List of the top 10 GO terms functionally enriched in the network of 13 genes significantly over expressed in MIAPaCa2 and at least one other pancreatic ductal adenocarcinoma (PDAC) cell lines (BxPC3 and CFPAC-1) investigated

GO term	GO description	FDR
GO:0051726	Regulation of cell cycle	8.16e-14
GO:0007049	Cell cycle	2.82e-9
GO:0000079	Regulation of cyclin-dependent protein serine/threonine kinases	4.89e-9
GO:0045786	Negative regulation of the cell cycle	4.65e-8
GO:0010212	Response to ionizing radiation	5.03e-8
GO:1903047	Mitotic cell cycle process	6.35e-8
GO:0022402	Cell cycle process	6.35e-8
GO:0030330	DNA-damage response	1.69e-7
GO:0044773	Mitotic DNA-damage checkpoint	4.42e-7
GO:0006259	DNA metabolic process	4.58e-7

One particularly striking change was in transcripts involved in the cellular response to stalled DNA-replication forks. This includes three of the four genes (*HUS1*,*RAD1*,*RAD17*) of the 9-1-1 check point complex clamp.^35^ Previous literature reports have shown that depletion of either *RAD9*^36^ or *RAD17*^37^ sensitizes HeLa and PDAC cells, respectively, to gemcitabine. Furthermore, other studies have identified that both *RAD9* and *HUS1* play an important role in the sensitivity of pancreatic cancer cells to gemcitabine.^36^ Together, these data demonstrate the importance of this signalling complex in the cellular response to gemcitabine and suggest that although the mode of action of 1-(*S*,*R*_p_) is distinct from gemcitabine, DNA-replication stress is central to the mechanism of action. Also upregulated were two of the three genes (*MRE11*, *NBN*) that form the MRE11/RAD50/NBS1 (MRN) complex, which plays a critical role in sensing DNA-double-strand breaks and initiating double-strand break repair by homologous recombination and non-homologous end joining, as well as activation of cell cycle checkpoints.^38^ This complex has also been demonstrated to play an important role in the cellular response to stalled replication forks after treatment of cancer cells with nucleoside analogues including gemcitabine.^39^ One of the major cellular responses to blocked DNA synthesis is activation of the S-phase DNA-damage response as discussed earlier and reviewed by Ewald *et al*.^40^ This results in inhibition of initiation and firing of replication forks consistent with our observation of S-phase cell cycle arrest.

Another key transcriptional change observed is strong induction of both the DNA-damage response protein *GADD45A* and the cyclin-dependent kinase inhibitor *CDKN1A* (p21). This is consistent with our observation of genotoxic stress discussed later in the text. Furthermore, *GADD45A* has previously been linked to chemoresistance in a number of cancer types including melanoma^41^ and glioblastoma^42^ but, to the best of our knowledge its role in the response of pancreatic cancer cells to nucleoside analogues has not been evaluated. Both *GADD45A* and *CDKN1A* are often considered to be downstream of, and transcriptionally activated by, p53^43^. Furthermore, within the network of 13 genes in total, 7 (*CDK2*, *MDM2*, *CASP3*, *CDKN1A*, *GADD45A*, *CCNG2*, *CCNE1*) were identified as being related to p53 signalling (Kyoto Encyclopedia of Genes and Genomes pathway HSA04115). Of these, four (*CDK2*, *GADD45A*, *CDKN1A*, *CCNE1*) were also represented in the reactome pathway TP53, which regulates transcription of cell cycle genes (HAS-6791312) and four (*CDK2*, *MDM2*, *CDKN1A*, *CCNE1*0) in the p53-dependent G1 DNA-damage response (HSA-69563). All PDAC cell lines used in this study contain inactivating mutations in p53, a fact that was confirmed directly by sequencing. Therefore, transcriptional activation of these genes must be through a mechanism that is independent of p53. Several possible mechanisms have been reported; indeed, it has been shown that some chemical agents including the alkylating agent methyl methane sulfonate and ultraviolet radiation appear to activate *GADD45A* via a mechanism that is at least partly p53 independent, involving the transcription factors Oct-1 and Nuclear transcription factor Y subunit alpha.^44^ Interestingly, overexpression of *GADD45A* has been linked to a poor prognosis in patients with PDAC,^45^ suggesting that this gene is important in PDAC cell phenotype and disease progression.

To further study the possible involvement of *TP53* in the cellular response to 1-(*S*,*R*_p_) we investigated the same set of 13 transcripts in HCT116 wild-type and *TP53* null cell lines. There was no difference in the observed transcriptional response (Supplementary Fig. S5A). In contrast, there was a small but statistically significant difference (*P* < 0.05) in the response of wild-type and p53 mutant HCT116 cells to 1-(*S*,*R*_p_). The modelled IC_50_ value of 1-(*S*,*R*_p_) in p53 wild-type and null cell lines were 11.9 (6.9–20.0) and 35.4 (21.8–58.9) μM, respectively (Supplementary Fig. S5B), suggesting that response to 1-(*S*,*R*_p_) may be at least partially dependent on p53. To further investigate p53-independent regulation of 1-(*S*,*R*_p_) responsive genes, the expression of the homologues p63 and p73 were investigated. Interestingly, treatment of PDAC cells with 1-(*S*,*R*_p_) resulted in induction of p73 but not p63 (Supplementary Fig. S6A). The levels of p73 transcript were also upregulated in HCT116 p53 knockout cells (Supplementary Fig. S6B) further supporting a compensatory role for this transcription factor in the response of cells to 1-(*S*,*R*_p_). The significance of these results is unclear; p73 can transactivate p53-regulated promoters and there is also evidence that in p53-deficient PDAC cell lines that sensitivity to gemcitabine can be enhanced through mechanisms that are dependent on both p63 and p73 transcription factor activity^46–49^. However, to the best of our knowledge there is no clear evidence linking p73 to the transcriptional regulation of either *GADD45A* or *CDKN1A*. We speculate that in PDAC cell lines, p73 may be able to partially compensate for the lack of p53-dependent induction of *GADD45A* and *CDKN1A* and that this is important in the cellular response to agents that cause replication fork and genotoxic stress.

### 1-*(S*,*R*_p_) induces DNA-strand breaks in newly synthesized genomic DNA

**Fig. 6:**
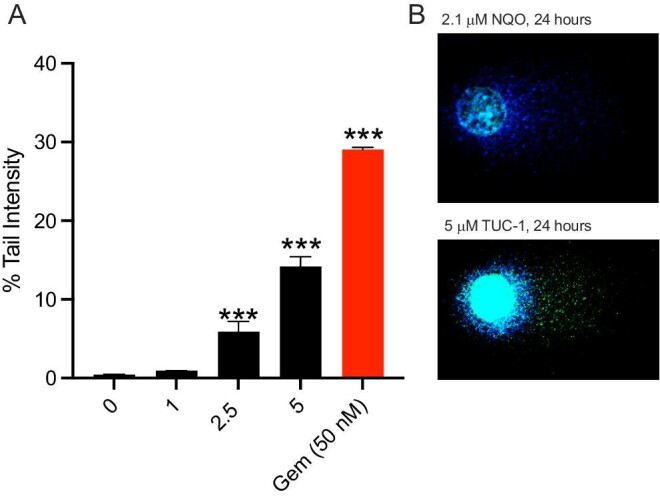
Compound 1-(*S*,*R*_p_) induced DNA-single-strand breaks in recently replicated genomic DNA. (A) Concentration-dependent increase in single-stranded DNA breaks following treatment with 1-(*S*,*R*_p_) (0–5 μM, 24 h) as assessed by the comet assay. (B) Pulse labelling with EdU (green fluorescence) prior to treatment confirms that DNA-strand breaks occur in recently replicated DNA following treatment with 1-(*S*,*R*_p_) (5 μM, 24 h) but not when treated with NQO a direct acting genotoxic chemical, where only non-EdU labelled DNA counterstained with Hoechst (blue fluorescence) is visible in the comet tail. The results represent the mean of three independent biological experiments (±SD, *n* = 3), *** significantly different from untreated control (*P* < 0.001, 1-way ANOVA followed by a *post hoc* Dunnett's *t*-test).

**Fig. 7:**
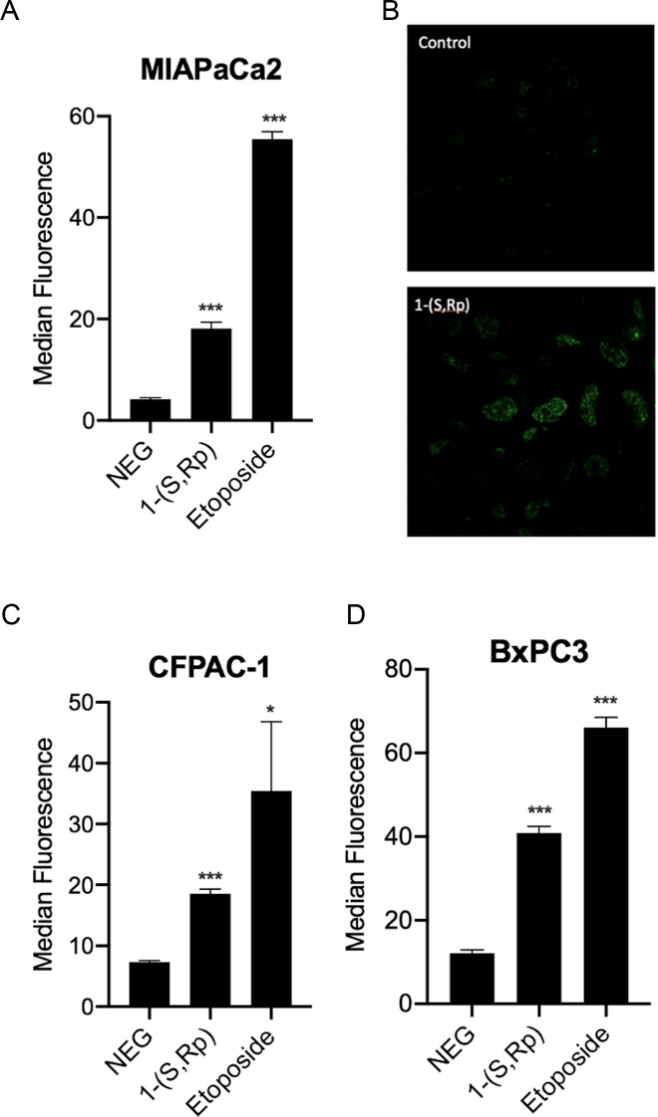
Compound 1-(*S*,*R*_p_) (10 μM, 24 h) induces double-stranded DNA breaks as assessed by gamma-H2AX phosphorylation in pancreatic ductal adeno carcinoma cells. (A) and (B) MIAPaCa2 cells as assessed by flow cytometry and confocal microscopy, respectively. (C) CFPAC-1 and (D) BxPC3 cells as assessed by flow cytometry. Etoposide (5 μM, 24 h) was used as a positive control. The results represent the mean of three independent biological experiments (±SD, *n* = 3). * and *** Statistically significantly different from control, *P* < 0.05 and 0.001, respectively (1-way ANOVA followed by a *post hoc* Dunnett's *t*-test).

To investigate whether the cell cycle and transcriptomic changes related to DNA-damage and DNA-replication fork stress are manifested as biochemical changes, we quantified the level of single and double DNA-strand breaks in cells using the alkaline comet assay and γH2AX staining, respectively. Consistent with DNA-strand breaks being important in the mode of action, treatment of MIAPaCa2 with 1-(*S*,*R*_p_) (0–5 μM, 24 h) resulted in a statistically significant (*P* < 0.001) concentration-dependent increase in the numbers of DNA-strand breaks as assessed by the percentage of DNA in the comet tail (Fig. 6A). Mean percentage tail DNA values were: 0.38 ± 0.11%, 0.93 ± 0.02%, 5.90 ± 1.3% and 14.2 ± 1.25% for cells treated with 0, 1, 2.5 and 5 μM, respectively. Consistent with previous studies,^50^^,^^51^ gemcitabine (50 nM, 24 h) also induced DNA-strand breaks (Fig. 6A). Next, to investigate whether DNA-strand breaks were occurring in newly synthesized DNA, the comet assay was repeated in cells that were pulse labelled with the fluorescent base EdU. When cells were treated with 1-(*S*,*R*_p_) (5 μM, 24 h) it was clear that the DNA present in the comet tail was labelled with EdU (Fig. 6B) confirming DNA-strand break formation in recently synthesized DNA consistent with replication fork stress. As a negative control, for cells treated with the direct acting genotoxin NQO (2.1 μM, 24 h) there was no evidence of EdU-labelled DNA in the comet tail (Fig. 6B). There was also evidence that treatment of all three PDAC cells lines with 1-(*S*,*R*_p_) (10 μM, 24 h) induced double DNA-strand breaks as assessed by phosphorylation of gamma-H2AX using both flow cytometry and confocal microscopy (Fig. 7). In addition to a marker of double-stranded DNA breaks, it has also been demonstrated that phosphorylation of γH2AX also occurs in response to stalled DNA-replication forks, including those induced by gemcitabine; this has been linked to S-phase checkpoint activation^50^ and recruitment of signalling molecules, including MRE11 and RAD51 to damaged DNA forks.^52^ Previous studies have also shown that the MRN complex regulates resistance of cancer cells to other nucleoside analogues where it is involved in the cellular response to stalled DNA-replication forks, but it was not clear whether this was directly related to DNA-strand breaks or via other mechanisms.^39^

### 1-(*S*,*R*_p_) induces replication fork arrest and activation of check point kinases

**Fig. 8:**
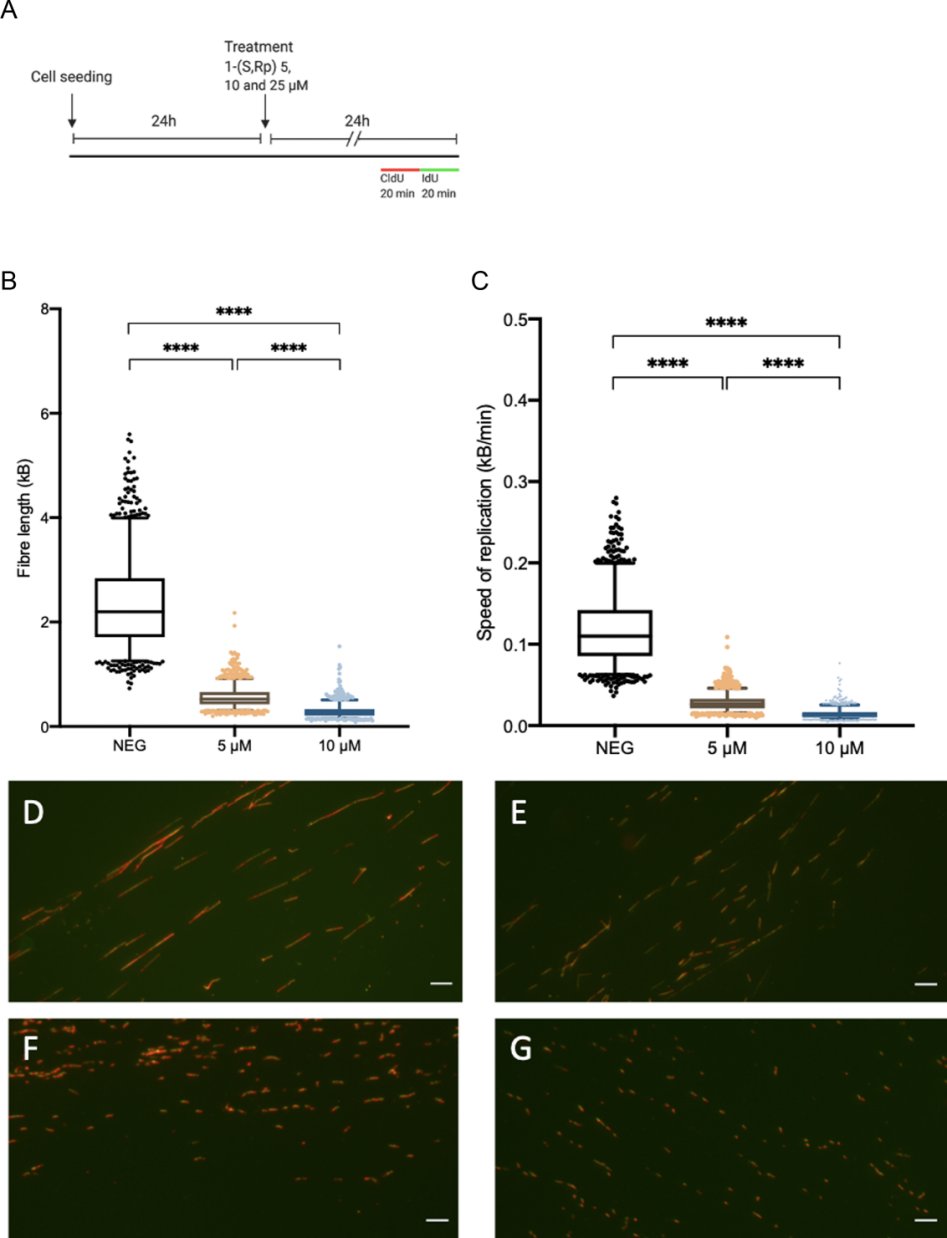
Compound 1-(*S*,*R*_p_) inhibits DNA replication as assessed by single molecule DNA-fibre fluorography analysis. (A) Experimental design: Cells were seeded at a density of 3 × 10^5^ cells/mL and allowed to attach for 24 h before treatment with 1-(*S*,*R*_p_) (5, 10 and 25 μM) for 24 h before labelling with CldU (red) and IdU (green). (B) Total fibre length (red + green label) in kB and (C) Replication rate Kb/min are inhibited in a concentration-dependent manner by 1-(*S*,*R*_p_). Box whisker plots show the mean (horizontal bar), interquartile range (box) and 5 and 95 percentile range (whisker) with values outside that range plotted as individual points. The results represent the mean of three independent experiments (*n* = 3) with 1072, 1406 and 1189 individual DNA fibres analysed in control, 5 and 10 μM treatments, respectively. Treatment with 25 μM 1-(*S*,*R*_p_) resulted in complete inhibition of DNA fibres that were not quantified. *** Statistically significant (*P* < 0.0001) as assessed by a 1-way ANOVA followed by a *post hoc* Dunnett's *t*-test. Representative images from (D) Control, (E) 5 μM, (F) 10 μM and (G) 25 μM 1-(*S*,*R*_p_) treated cells. Scale bar 10 μm and fibre length in micrometres was converted to kB of DNA as described in the materials and methods.

To directly investigate the effect on active DNA replication and down-stream signalling events we used single molecule DNA-fibre fluorography to study replication fork dynamics. Our experimental design is summarized in Fig. 8A. Treatment with 1-(*S*,*R*_p_) (5, 10, 25 μM) for 24 h resulted in concentration-dependent inhibition of DNA replication and evidence of stalled replication forks. Fibre length in micrometres was converted to kB of DNA as described previously.^29^ In untreated control MIAPaCa2 cells, mean fibre length was 2.34 ± 0.8 kB (range 0.73–5.60). Cells treated with 1-(*S*,*R*_p_) had statistically significantly shorter fibres (*P* < 0.001). Following treatment with 5 and 10 μM 1-(*S*,*R*_p_), mean fibre lengths were 0.56 ± 0.20 (range 0.19–2.17) and 0.28 ± 0.12 (range 0.08–1.53) kB, respectively (Fig. 8B). Representative images of DNA-fibres from each experimental condition are shown in Fig. 8D–G. Treatment with 25 μM 1-(*S*,*R*_p_) resulted in complete arrest of fibre formation (Fig. 8G). As expected, treatment also resulted in a concentration-dependent decrease in the speed of DNA replication (Fig. 8C). Calculated DNA-replication rates were 0.12 ± 0.04, 0.028 ± 0.01 and 0.014 ± 0.006 kB/minute in control, 5 and 10 μM treatments, respectively.

These data confirm the effect of 1-(*S*,*R*_p_) on DNA replication as quantified by incorporation of EdU as described earlier. Furthermore, consistent with this and the transcriptional changes described earlier we also observed that 1-(*S*,*R*_p_) treatment results in phosphorylation of the checkpoint kinases CHEK1 (Ser 345) and CHEK2 (Thr 68), replication protein A (RPA) and gamma-H2AX (Ser 139) as assessed by western blotting (Supplementary Fig. S7). checkpoint kinase (CHK)1 and CHK2 are critical regulators in the coordinated cellular response to DNA damage and stalled replication forks^53^ and are important in the response of cancer cells to gemcitabine. In fact, previous studies have shown that CHK1 kinase inhibitors sensitize pancreatic cancer cells to the toxicity of gemcitabine,^54–58^ a strategy that has shown promise in clinical trials.^59^ Together these data strongly support our hypothesis that 1-(*S*,*R*_p_) stalls DNA-replication forks activating downstream signalling pathways, ultimately resulting in S-phase arrest and cell death by apoptosis.

Our previous SAR studies have shown that both regiochemistry^23^ and linker length^22^ are key determinants of cytotoxicity for 1-(*S*,*R*_p_). These observations, coupled with our evidence that 1-(*S*,*R*_p_) appears not to be directly incorporated into genomic DNA via phosphorylation,^23^ while still inducing both single and double DNA-strand breaks as shown here, suggests a specific cellular target associated with DNA-replication machinery. On one level, it could be argued that this would be at the expense of a non-specific mechanism of toxicity, such as redox cycling of the iron and generation of reactive oxygen species, a mechanism that has been suggested for both the parent ferrocene compound^60^^,^^61^ as well as other ferrocene analogues including ferrocifens^62^^,^^63^ and its derivatives.^64^ However, on the other hand, and in support of a possible role of redox activity, a ruthenium analogue of 1-(*S*,*R*_p_), which shows less reversible electrochemistry, and whose oxidized form is less accessible in biological systems, is not toxic to the MIAPaCa2 cell line.^21^ Therefore a role for the iron in the toxicity of 1-(*S*,*R*_p_) is a distinct possibility, e.g. via generation of localized redox activity at a cellular target as part of the mechanism of action. Future studies to investigate transcriptional changes in oxidative stress genes would help to address this. Interestingly, DNA polymerases are known to contain iron-sulphur clusters and it is becoming increasingly apparent that they are involved in redox regulation of polymerase functions.^65^^,^^66^ It is possible therefore, that modification of the local redox environment by 1-(*S*,*R*_p_) affects DNA polymerase activity and that this plays a role in the molecular mode of action. Future *in silico* and biochemical studies to investigate direct docking with DNA polymerase would be informative. However, we cannot exclude at this point other cellular targets linked to DNA processing, e.g. inhibition of DNA topoisomerase or depletion of the nucleotide pool as possible mechanisms of 1-(*S*,*R*_p_) toxicity.

## Conclusion

**Fig. 9:**
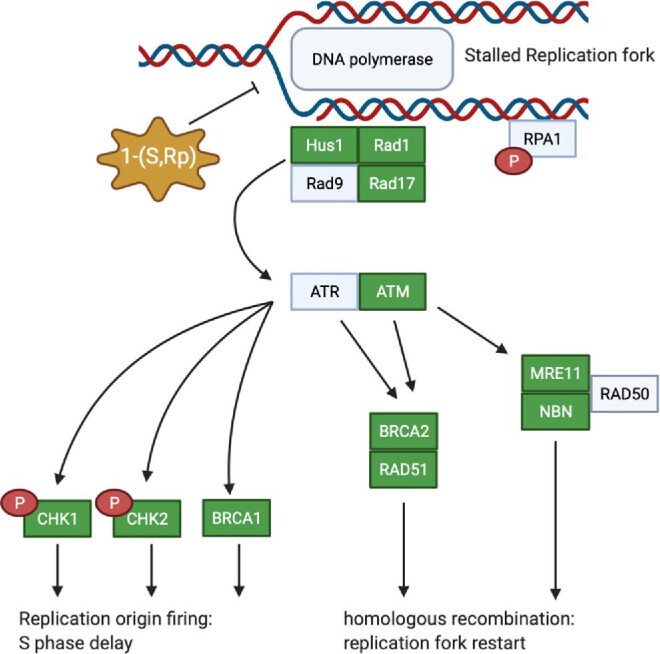
Compound 1-(*S*,*R*_p_) stalls DNA replication in pancreatic ductal adenoma carcinoma cells resulting in activation of downstream signalling pathways and replication fork arrest. Highlighted in green are genes of this pathway that are statistically significantly transcriptionally activated in response to treatment with 1-(*S*,*R*_p_). These include three out of the four genes involved in formation of the ‘9-1-1 checkpoint complex clamp’ (HUS1, Rad1 and Rad17) and two out of the three genes that form the MRN complex (MRE11 and NBN). Also detected experimentally and shown as red circles was phosphorylation of RPA1 and both checkpoint kinases 1 and 2. Diagram created in Biorender, www.biorender.com.

In study, we have shown that the organometallic ferronucleoside 1-(*S*,*R*_p_) has cytotoxic activity in multiple pancreatic cancer cells and that the mode of action is inhibition of DNA replication, resulting in replication fork stress, cell cycle arrest and apoptosis. Our data point to a mechanism of action of 1-(*S*,*R*_p_) that is distinct from that of establised organic nucleoside analogues such as 5-fluorouracil and gemcitabine currently used to treat PDAC. Our data further highlight that synthetic nucleoside analogues are cytotoxic to cells through multiple modes of action. Furthermore, the toxicity and mechanism of action of 1-(*S*,*R*_p_) is at least partially independent of p53 transcription factor activity. Cytotoxicity is dependent on both the linker length and regiochemistry of 1-(*S*,*R*_p_) as well as the presence of a redox-active metal centre. We therefore hypothesize that the mechanism of action is through interaction with, and local redox modification of specific cellular targets. Based on the observation of inhibition of, DNA replication, induction of DNA breaks specifically in recently synthesized DNA and replication fork arrest, we hypothesize that the cellular target is the DNA-replication machinery itself (summarized in Fig. 9). In conclusion, because of its novel mode of action, potency and activity in gemcitabine-resistant cells, 1-(*S*,*R*_p_) is a promising candidate molecule for development of new treatments for PDAC.

## Supplementary Material

mfac041_Supplemental_Files

## Data Availability

The data underlying this article are available in the article and in its online supplementary material.

## References

[1] Allemani C., Matsuda T., Di Carlo V., Harewood R., Matz M., Niksic M., Bonaventure A., Valkov M., Johnson C. J., Esteve J., Ogunbiyi O. J., Azevedo E. S. G., Chen W. Q., Eser S., Engholm G., Stiller C. A., Monnereau A., Woods R. R., Visser O., Lim G. H., Aitken J., Weir H. K., Coleman M. P., C W. Group, Global surveillance of trends in cancer survival 2000-14 (CONCORD-3): analysis of individual records for 37 513 025 patients diagnosed with one of 18 cancers from 322 population-based registries in 71 countries, The Lancet, 2018, 391 (10125), 1023–1075. 10.1016/S0140-6736(17)33326-3 PMC5879496 29395269

[2] Conroy T., Desseigne F., Ychou M., Bouche O., Guimbaud R., Becouarn Y., Adenis A., Raoul J. L., Gourgou-Bourgade S., de la Fouchardiere C., Bennouna J., Bachet J. B., Khemissa-Akouz F., Pere-Verge D., Delbaldo C., Assenat E., Chauffert B., Michel P., Montoto-Grillot C., Ducreux M., Unicanc G. T. D., Intergrp P., FOLFIRINOX versus gemcitabine for metastatic pancreatic cancer, N. Engl. J. Med., 2011, 364 (19), 1817–1825. 21561347 10.1056/NEJMoa1011923

[3] Pacheco-Barcia V., France T., Zogopoulos G., Bouganim N., Donnay O., Alcindor T., Solis R. M., Guo K., Martin E., Colomer R., Asselah J., Gemcitabine plus nab-paclitaxel versus modified FOLFIRINOX as first line chemotherapy in metastatic pancreatic cancer: a comparison of toxicity and survival, Ann. Oncol., 2018, 29 (Suppl 5), v46.

[4] Williet N., Saint A., Pointet A. L., Tougeron D., Pernot S., Pozet A., Bechade D., Trouilloud I., Lourenco N., Hautefeuille V., Locher C., Desrame J., Artru P., Thirot Bidault A., Le Roy B., Pezet D., Phelip J. M., Taieb J., Folfirinox versus Gemcitabine/Nab-Paclitaxel as first-line therapy in patients with metastatic pancreatic cancer: a comparative propensity score study, Therap. Adv. Gastroenterol., 2019, 12, 175628481987866. 10.1177/1756284819878660 PMC6764033 31598136

[5] Cho I., Kang H., Jo J., Lee H., Chung M., Park J., Park S., Song S., Park M., An C., Jung S., Bang S., FOLFIRINOX versus gemcitabine plus Nab-Paclitaxel for treatment of metastatic pancreatic cancer: a single-center cohort study, Ann. Oncol., 2018, 29 (Suppl 5), v45.

[6] Ramesh R., Reddy D S., Quest for novel chemical entities through incorporation of silicon in drug Scaffolds, J. Med. Chem., 2018, 61 (9), 3779–3798. 29039662 10.1021/acs.jmedchem.7b00718

[7] Karges J., Stokes R. W., Cohen S M., Metal complexes for therapeutic applications, Trends Chem., 2021, 7, 523–534. 10.1016/j.trechm.2021.03.006 PMC9374106 35966501

[8] Jaouen G., Vessieres A., Top S., Ferrocifen type anti cancer drugs, Chem. Soc. Rev., 2015, 44 (24), 8802–8817. 26486993 10.1039/c5cs00486a

[9] Patra M., Gasser G., The medicinal chemistry of ferrocene and its derivatives, Nat. Rev. Chem., 2017, 1 (9), 0066.

[10] Gasser G., Metzler-Nolte N., The potential of organometallic complexes in medicinal chemistry, Curr. Opin. Chem. Biol., 2012, 16 (1-2), 84–91. 22366385 10.1016/j.cbpa.2012.01.013

[11] Kopfmaier P., Kopf H., Neuse E W., Ferrocenium salts – the 1st antineoplastic iron compounds, Angew Chem Int Edit 1984, 23 (6), 456–457.

[12] Patra M., Ingram K., Pierroz V., Ferrari S., Spingler B., Keiser J., Gasser G., Ferrocenyl derivatives of the anthelmintic praziquantel: design, synthesis, and biological evaluation, J. Med. Chem., 2012, 55 (20), 8790–8798. 23005702 10.1021/jm301077m

[13] Chohan Z H. , Synthesis of organometallic-based biologically active compounds: in vitro antibacterial, antifungal and cytotoxic properties of some sulfonamide incorporated ferrocences, J. Enzyme. Inhib. Med. Chem., 2009, 24 (1), 169–175. 18608785 10.1080/14756360801948766

[14] Rubbiani R., Blacque O., Gasser G., Sedaxicenes: potential new antifungal ferrocene-based agents? Dalton. Trans., 2016, 45 (15) 6619–6626. 26964501 10.1039/c5dt04231c

[15] Wani W. A., Jameel E., Baig U., Mumtazuddin S., Hun L T., Ferroquine and its derivatives: new generation of antimalarial agents. Eur. J. Med. Chem., 2015, 101, 534–551. 26188909 10.1016/j.ejmech.2015.07.009 PMC7115395

[16] Top S., Vessieres A., Leclercq G., Quivy J., Tang J., Vaissermann J., Huche M., Jaouen G., Synthesis, biochemical properties and molecular modelling studies of organometallic specific estrogen receptor modulators (SERMs), the ferrocifens and hydroxyferrocifens: evidence for an antiproliferative effect of hydroxyferrocifens on both hormone-dependent and hormone-independent breast cancer cell lines, Chemistry, 2003, 9 (21), 5223–5236. 14613131 10.1002/chem.200305024

[17] Bruyere C., Mathieu V., Vessieres A., Pigeon P., Top S., Jaouen G., Kiss R., Ferrocifen derivatives that induce senescence in cancer cells: selected examples, J. Inorg. Biochem., 2014, 141, 144–151. 25261808 10.1016/j.jinorgbio.2014.08.015

[18] Carmona-Negron J. A., Santana A., Rheingold A. L., Melendez E., Synthesis, structure, docking and cytotoxic studies of ferrocene-hormone conjugates for hormone-dependent breast cancer application, Dalton Trans, 2019, 48, (18), 5952–5964. 30507996 10.1039/c8dt01856a

[19] Romeo G., Chiacchio U., Corsaro A., Merino P., Chemical synthesis of heterocyclic-sugar nucleoside analogues, Chem Rev., 2010, 110 (6), 3337–3370. 20232792 10.1021/cr800464r

[20] Nguyen H. V., Sallustrau A., Balzarini J., Bedford M. R., Eden J. C., Georgousi N., Hodges N. J., Kedge J., Mehellou Y., Tselepis C., Tucker J. H. R., Organometallic nucleoside analogues with ferrocenyl linker groups: synthesis and cancer cell line studies, J. Med. Chem., 2014, 57 (13), 5817–5822. 24905419 10.1021/jm500246h

[21] Ismail M. K., Armstrong K. A., Hodder S. L., Horswell S. L., Male L., Nguyen H. V., Wilkinson E. A., Hodges N. J., Tucker J. H. R., Organometallic nucleoside analogues: effect of the metallocene metal atom on cancer cell line toxicity, Dalton Trans., 2020, 49 (4), 1181–1190. 31897458 10.1039/c9dt04174e

[22] Kedge J. L., Nguyen H. V., Khan Z., Male L., Ismail M. K., Roberts H. V., Hodges N. J., Horswell S. L., Mehellou Y., Tucker J. H. R., Organometallic nucleoside analogues: effect of hydroxyalkyl linker length on cancer cell line toxicity, Eur. J. Inorg. Chem., 2017 (2), 466–476.

[23] Ismail M., Khan Z., Rana M., Horswell S., Male L., Perotti A., Romero-Canelon I., Wilkinson E., Hodges N., Nguyen H., Tucker J. H. R., Effect of regiochemistry and methylation on the anticancer activity of a ferrocene-containing organometallic nucleoside analogue, ChemBioChem. 2020, 21 (917), 2487–2494. 32255248 10.1002/cbic.202000124

[24] Bunz F., Dutriaux A., Lengauer C., Waldman T., Zhou S., Brown J. P., Sedivy J. M., Kinzler K. W., Vogelstein B., Requirement for p53 and p21 to sustain G2 arrest after DNA damage, Science, 1998, 282 (5393), 1497–1501. 9822382 10.1126/science.282.5393.1497

[25] Kato S., Han S. Y., Liu W., Otsuka K., Shibata H., Kanamaru R., Ishioka C., Understanding the function-structure and function-mutation relationships of p53 tumor suppressor protein by high-resolution missense mutation analysis, Proc Natl Acad Sci USA, 2003, 100 (14), 8424–8429. 12826609 10.1073/pnas.1431692100 PMC166245

[26] Stockert J. C., Horobin R. W., Colombo L. L., Blazquez-Castro A., Tetrazolium salts and formazan products in cell biology: viability assessment, fluorescence imaging, and labeling perspectives, Acta Histochem, 2018, 120 (3), 159–167. 29496266 10.1016/j.acthis.2018.02.005

[27] Kim K. H., Sederstrom J M., Assaying cell cycle status using flow cytometry, Curr. Protoc. Mol. Biol., 2015, 111, 28 6 1-6 11. 10.1002/0471142727.mb2806s111 PMC4516267 26131851

[28] Henry-Mowatt J., Jackson D., Masson J. Y., Johnson P. A., Clements P. M., Benson F. E., Thompson L. H., Takeda S., West S. C., Caldecott K. W., XRCC3 and Rad51 modulate replication fork progression on damaged vertebrate chromosomes, Mol. Cell., 2003, 11 (4), 1109–1117. 12718895 10.1016/s1097-2765(03)00132-1

[29] Nieminuszczy J., Schwab R. A., Niedzwiedz W., The DNA fibre technique – tracking helicases at work, Methods, 2016, 108, 92–98. 27102626 10.1016/j.ymeth.2016.04.019

[30] Burris H. A. 3rd, Moore M. J., Andersen J., Green M. R., Rothenberg M. L., Modiano M. R., Cripps M. C., Portenoy R. K., Storniolo A. M., Tarassoff P., Nelson R., Dorr F. A., Stephens C. D., Von Hoff D D., Improvements in survival and clinical benefit with gemcitabine as first-line therapy for patients with advanced pancreas cancer: a randomized trial, J. Clin. Oncol., 1997, 15 (6), 2403–2413. 9196156 10.1200/JCO.1997.15.6.2403

[31] Galanski M. , Recent developments in the field of anticancer platinum complexes, Recent. Pat. Anticancer. Drug. Discov., 2006, 1 (2), 285–295. 18221042 10.2174/157489206777442287

[32] Huang L., Wong Y. P., Cai Y. J., Lung I., Leung C. S., Burd A., Low-dose 5-fluorouracil induces cell cycle G2 arrest and apoptosis in keloid fibroblasts, Br. J. Dermatol., 2010, 163 (6), 1181–1185. 20633010 10.1111/j.1365-2133.2010.09939.x

[33] Cappella P., Tomasoni D., Faretta M., Lupi M., Montalenti F., Viale F., Banzato F., D'Incalci M., Ubezio P., Cell cycle effects of gemcitabine, Int. J. Cancer., 2001, 93 (3), 401–408. 11433406 10.1002/ijc.1351

[34] Szklarczyk D., Franceschini A., Wyder S., Forslund K., Heller D., Huerta-Cepas J., Simonovic M., Roth A., Santos A., Tsafou K. P., Kuhn M., Bork P., Jensen L. J., von Mering C., STRING v10: protein-protein interaction networks, integrated over the tree of life, Nucleic. Acids. Res., 2015, 43 (Database issue), D447–D452. 25352553 10.1093/nar/gku1003 PMC4383874

[35] Parrilla-Castellar E. R., Arlander S. J., Karnitz L., Dial 9-1-1 for DNA damage: the Rad9-Hus1-Rad1 (9-1-1) clamp complex. DNA Repair (Amst), 2004, 3 (8-9), 1009–1014. 15279787 10.1016/j.dnarep.2004.03.032

[36] Karnitz L. M., Flatten K. S., Wagner J. M., Loegering D., Hackbarth J. S., Arlander S. J., Vroman B. T., Thomas M. B., Baek Y. U., Hopkins K. M., Lieberman H. B., Chen J., Cliby W. A., Kaufmann S H., Gemcitabine-induced activation of checkpoint signaling pathways that affect tumor cell survival, Mol. Pharmacol., 2005, 68 (6), 1636–1644. 16126823 10.1124/mol.105.012716

[37] Fredebohm J., Wolf J., Hoheisel J. D., Boettcher M., Depletion of RAD17 sensitizes pancreatic cancer cells to gemcitabine, J. Cell. Sci. 2013, 126 (Pt 15), 3380–3389. 23687379 10.1242/jcs.124768

[38] Lamarche B. J., Orazio N. I., Weitzman M D., The MRN complex in double-strand break repair and telomere maintenance, FEBS. Lett., 2010, 584 (17), 3682–3695. 20655309 10.1016/j.febslet.2010.07.029 PMC2946096

[39] Ewald B., Sampath D., Plunkett W., ATM and the Mre11-Rad50-Nbs1 complex respond to nucleoside analogue-induced stalled replication forks and contribute to drug resistance, Cancer. Res., 2008, 68 (19), 7947–7955. 18829552 10.1158/0008-5472.CAN-08-0971 PMC2631429

[40] Ewald B., Sampath D., Plunkett W., Nucleoside Analogs: molecular mechanisms signaling cell death, Oncogene., 2008, 27 (50), 6522–6537. 18955977 10.1038/onc.2008.316

[41] Liu J., Jiang G., Mao P., Zhang J., Zhang L., Liu L., Wang J., Owusu L., Ren B., Tang Y., Li W., Down-regulation of GADD45A enhances chemosensitivity in melanoma, Sci. Rep., 2018, 8 (1), 4111. 29515153 10.1038/s41598-018-22484-6 PMC5841426

[42] Wang H. H., Chang T. Y., Lin W. C., Wei K. C., Shin J W., GADD45A plays a protective role against Temozolomide treatment in glioblastoma cells, Sci. Rep., 2017, 7 (1), 8814. 28821714 10.1038/s41598-017-06851-3 PMC5562912

[43] Kastan M. B., Zhan Q., el-Deiry W. S., Carrier F., Jacks T., Walsh W. V., Plunkett B. S., Vogelstein B., Fornace A. J. Jr., A mammalian cell cycle checkpoint pathway utilizing p53 and GADD45 is defective in ataxia-telangiectasia, Cell., 1992, 71 (4), 587–597. 1423616 10.1016/0092-8674(92)90593-2

[44] Jin S., Fan F., Fan W., Zhao H., Tong T., Blanck P., Alomo I., Rajasekaran B., Zhan Q., Transcription factors Oct-1 and NF-YA regulate the p53-independent induction of the GADD45 following DNA damage, Oncogene, 2001, 20 (21), 2683–2690. 11420680 10.1038/sj.onc.1204390

[45] Hildesheim J., Fornace A. J. Jr., Gadd45a: an elusive yet attractive candidate gene in pancreatic cancer, Clin. Cancer. Res., 2002, 8 (8), 2475–2479. 12171872

[46] Ozaki T., Yu M., Yin D., Sun D., Zhu Y., Bu Y., Sang M., Impact of RUNX2 on drug-resistant human pancreatic cancer cells with p53 mutations, BMC Cancer., 2018, 18 (1), 309. 29558908 10.1186/s12885-018-4217-9 PMC5861661

[47] Acedo P., Fernandes A., Zawacka-Pankau J., Activation of TAp73 and inhibition of TrxR by verteporfin for improved cancer therapy in TP53 mutant pancreatic tumors, Future Sci. OA., 2019, 5 (2), FSO366. 30820346 10.4155/fsoa-2018-0082 PMC6391631

[48] Nakamura M., Sugimoto H., Ogata T., Hiraoka K., Yoda H., Sang M., Sang M., Zhu Y., Yu M., Shimozato O., Ozaki T., Improvement of gemcitabine sensitivity of p53-mutated pancreatic cancer MiaPaCa-2 cells by RUNX2 depletion-mediated augmentation of TAp73-dependent cell death, Oncogenesis, 2016, 5 (6), e233. 27294865 10.1038/oncsis.2016.40 PMC4945741

[49] Sang M., Nakamura M., Ogata T., Sun D., Shimozato O., Nikaido T., Ozaki T., Impact of RUNX2 gene silencing on the gemcitabine sensitivity of p53mutated pancreatic cancer MiaPaCa2 spheres, Oncol. Rep., 2018, 39 (6), 2749–2758. 29620279 10.3892/or.2018.6344

[50] Ewald B., Sampath D., Plunkett W., H2AX phosphorylation marks gemcitabine-induced stalled replication forks and their collapse upon S-Phase checkpoint abrogation, Mol. Cancer. Ther., 2007, 6 (4), 1239–1248. 17406032 10.1158/1535-7163.MCT-06-0633

[51] Sampath D., Rao V. A., Plunkett W., Mechanisms of apoptosis induction by nucleoside analogs. Oncogene 2003, 22 (56), 9063–9074. 14663485 10.1038/sj.onc.1207229

[52] Sirbu B. M., Couch F. B., Feigerle J. T., Bhaskara S., Hiebert S. W., Cortez D., Analysis of protein dynamics at active, stalled, and collapsed replication forks, Genes. Dev., 2011, 25 (12), 1320–1327. 21685366 10.1101/gad.2053211 PMC3127432

[53] Bartek J., Lukas J., Chk1 and Chk2 kinases in checkpoint control and cancer, Cancer Cell., 2003, 3 (5), 421–429. 12781359 10.1016/s1535-6108(03)00110-7

[54] Liang M., Zhao T., Ma L., Guo Y., CHK1 inhibition sensitizes pancreatic cancer cells to gemcitabine via promoting CDK-dependent DNA damage and ribonucleotide reductase downregulation, Oncol. Rep., 2018, 39 (3), 1322–1330. 29286153 10.3892/or.2017.6168

[55] Barnard D., Diaz H. B., Burke T., Donoho G., Beckmann R., Jones B., Barda D., King C., Marshall M., LY2603618, a selective CHK1 inhibitor, enhances the anti-tumor effect of gemcitabine in Xenograft tumor models, Invest. New. Drugs., 2016, 34 (1), 49–60. 26612134 10.1007/s10637-015-0310-y

[56] Koh S. B., Courtin A., Boyce R. J., Boyle R. G., Richards F. M., Jodrell D I., CHK1 inhibition synergizes with gemcitabine initially by destabilizing the DNA replication apparatus. Cancer Res., 2015, 75 (17), 3583–3595. 26141863 10.1158/0008-5472.CAN-14-3347

[57] Montano R., Thompson R., Chung I., Hou H., Khan N., Eastman A., Sensitization of human cancer cells to gemcitabine by the Chk1 inhibitor MK-8776: cell cycle perturbation and impact of administration schedule in vitro and in vivo, BMC Cancer, 2013, 13, 604. 24359526 10.1186/1471-2407-13-604 PMC3878047

[58] Venkatesha V. A., Parsels L. A., Parsels J. D., Zhao L., Zabludoff S. D., Simeone D. M., Maybaum J., Lawrence and T. S., Morgan M A., Sensitization of pancreatic cancer stem cells to gemcitabine by Chk1 inhibition, Neoplasia, 2012, 14 (6), 519–525. 22787433 10.1593/neo.12538 PMC3394194

[59] Calvo E., Braiteh F., Von Hoff D., McWilliams R., Becerra C., Galsky M. D., Jameson G., Lin J., McKane S., Wickremsinhe E. R., Hynes S. M., Bence Lin A., Hurt K., Richards D., Phase I study of CHK1 inhibitor LY2603618 in combination with gemcitabine in patients with solid tumors, Oncology-Basel, 2016, 91 (5), 251–260. 10.1159/000448621 27598338

[60] Wenzel M., Wu Y., Liss E., Neuse E W., Stability of ferricinium cations and their cytostatic effect, Z. Naturforsch. C J. Biosci., 1988, 43 (11-12), 963–966. 3245882

[61] Kopf-Maier P., Kopf H., Neuse E W., Ferricenium complexes: a new type of water-soluble antitumor agent, J. Cancer Res. Clin. Oncol. 1984, 108 (3), 336–340. 6511806 10.1007/BF00390468 PMC12253323

[62] Lu C., Heldt J. M., Guille-Collignon M., Lemaitre F., Jaouen G., Vessieres A., Amatore C., Quantitative analyses of ROS and RNS production in breast cancer cell lines incubated with Ferrocifens, ChemMedChem., 2014, 9 (6), 1286–1293. 24803138 10.1002/cmdc.201402016

[63] Vessieres A., Corbet C., Heldt J. M., Lories N., Jouy N., Laios I., Leclercq G., Jaouen G., Toillon R A., A ferrocenyl derivative of hydroxytamoxifen elicits an estrogen receptor-independent mechanism of action in breast cancer cell lines, J. Inorg. Biochem., 2010, 104 (5), 503–511. 20116857 10.1016/j.jinorgbio.2009.12.020

[64] Hagen H., Marzenell P., Jentzsch E., Wenz F., Veldwijk M. R., Mokhir A., Aminoferrocene-based prodrugs activated by reactive oxygen species, J. Med. Chem., 2012, 55 (2), 924–934. 22185340 10.1021/jm2014937

[65] Barton J. K., Silva R. M. B., O'Brien E., Redox chemistry in the genome: emergence of the [4Fe4S] cofactor in repair and replication, Annu. Rev. Biochem., 2019, 88, 163–190. 31220976 10.1146/annurev-biochem-013118-110644 PMC6590699

[66] Baranovskiy A. G., Siebler H. M., Pavlov Y. I., Tahirov T H., Iron-sulfur clusters in DNA polymerases and primases of eukaryotes, Methods Enzymol., 2018, 599, 1–20. 29746236 10.1016/bs.mie.2017.09.003 PMC5947875

