# Supplementary material for: A ferrocene-containing nucleoside analogue targets DNA replication in pancreatic cancer cells
Source: Metallomics. 2022 Jun 11;14(7):mfac041. doi: 10.1093/mtomcs/mfac041 (PMC9320222; doi:10.1093/mtomcs/mfac041)
Supplement: mfac041_Supplemental_Files [file mfac041_supplemental_files.zip › SupplFig1_pdf.pdf]

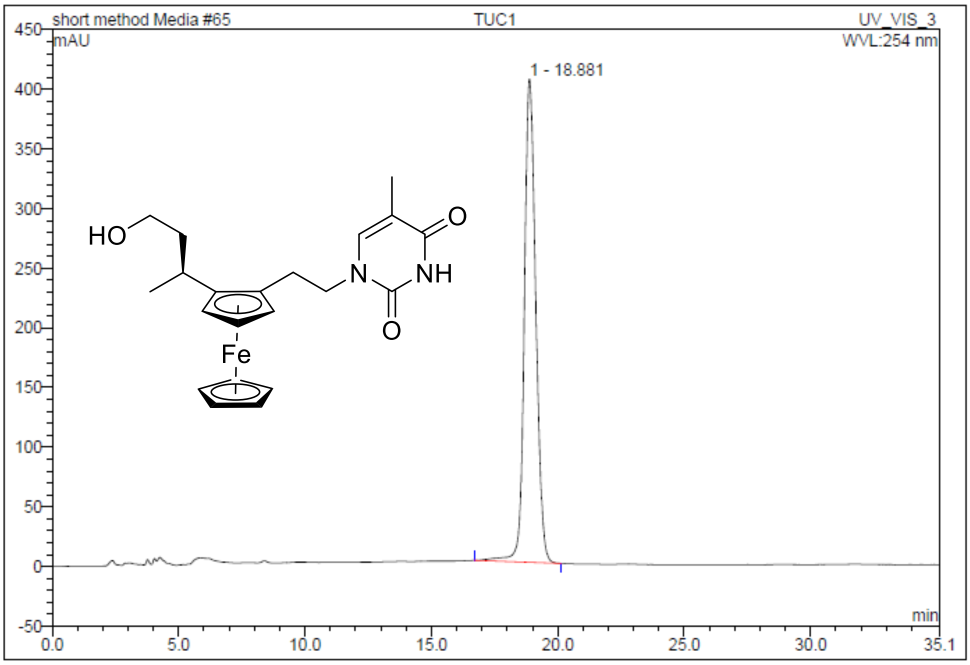


| No. | Ret.Time min | Peak Name | Height mAU | Area mAU*min | Rel.Area % | Amount | Type |
|---|---|---|---|---|---|---|---|
| 1 | 18.88 | n.a. | 405.397 | 213.749 | 100.00 | n.a. | BMB |
| **Total:** | | | 405.397 | 213.749 | 100.00 | 0.000 | |

**Figure S1**: Assessment of chiral purity by HPLC of ferro-nucleobase (S,Rp)-1-[α-Methyl-(3-(hydroxy)propyl)]-2-[(thyminyl)ethyl]-ferrocene, 1-***(S,R$_p$)***. Samples were eluted on a cellulose 1 column with a flow rate of 1 mL min$^{-1}$ using 40% MeCN in $H_2O$ as the solvent.
