# Supplementary material for: A ferrocene-containing nucleoside analogue targets DNA replication in pancreatic cancer cells
Source: Metallomics. 2022 Jun 11;14(7):mfac041. doi: 10.1093/mtomcs/mfac041 (PMC9320222; doi:10.1093/mtomcs/mfac041)
Supplement: mfac041_Supplemental_Files [file mfac041_supplemental_files.zip › SupplFig2_pdf.pdf]

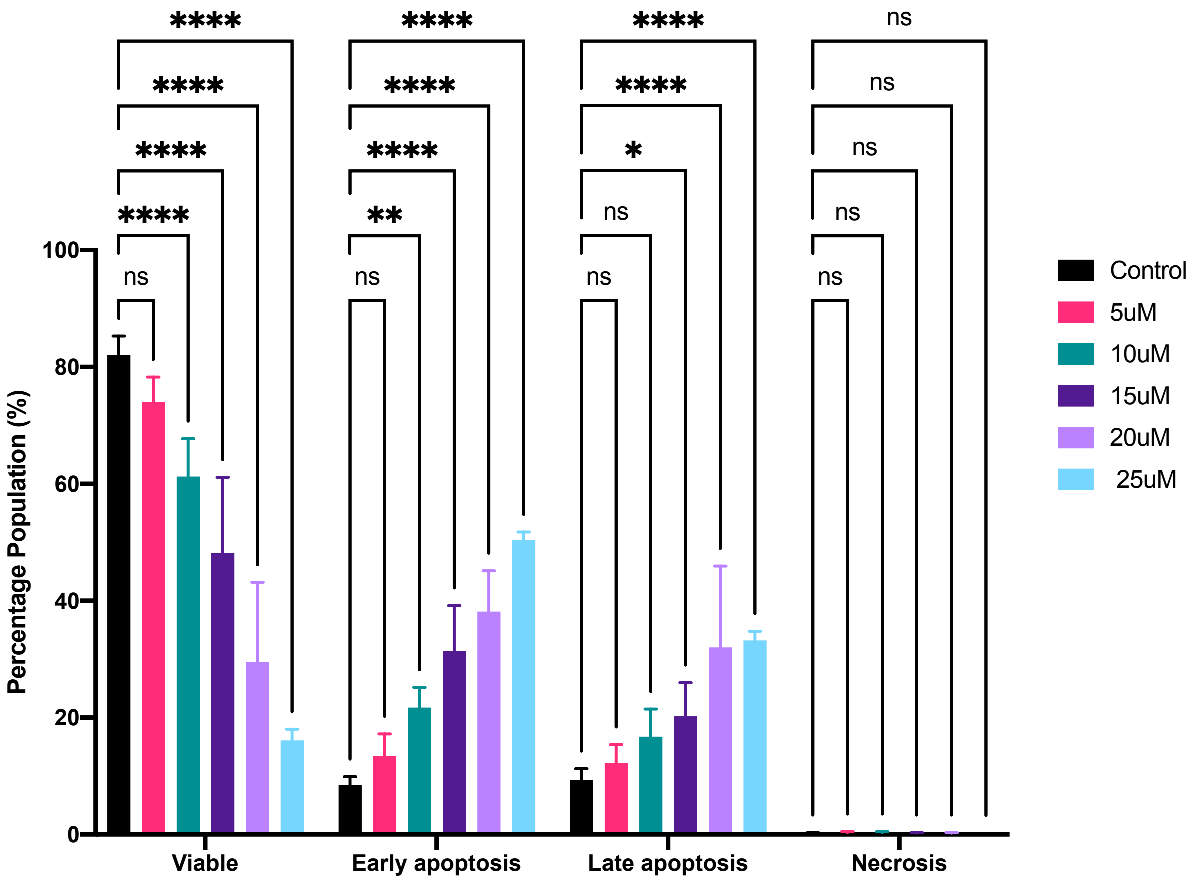

Figure S2: Induction of apoptosis as assessed by annexin and PI staining in MIAPaCa2 cells following treatment with 0-25 µM **1-(*S*,*R*$_p$)** for 72 hours. * and **** statistically significant $P<0.05$ and 0.0001 as assessed by a 2-way ANOVA followed by a *post-hoc* Dunnett's t-test. The results represent the mean of three independent experiments carried out in duplicate.
