# Supplementary material for: A ferrocene-containing nucleoside analogue targets DNA replication in pancreatic cancer cells
Source: Metallomics. 2022 Jun 11;14(7):mfac041. doi: 10.1093/mtomcs/mfac041 (PMC9320222; doi:10.1093/mtomcs/mfac041)
Supplement: mfac041_Supplemental_Files [file mfac041_supplemental_files.zip › SupplFig3_pdf.pdf]

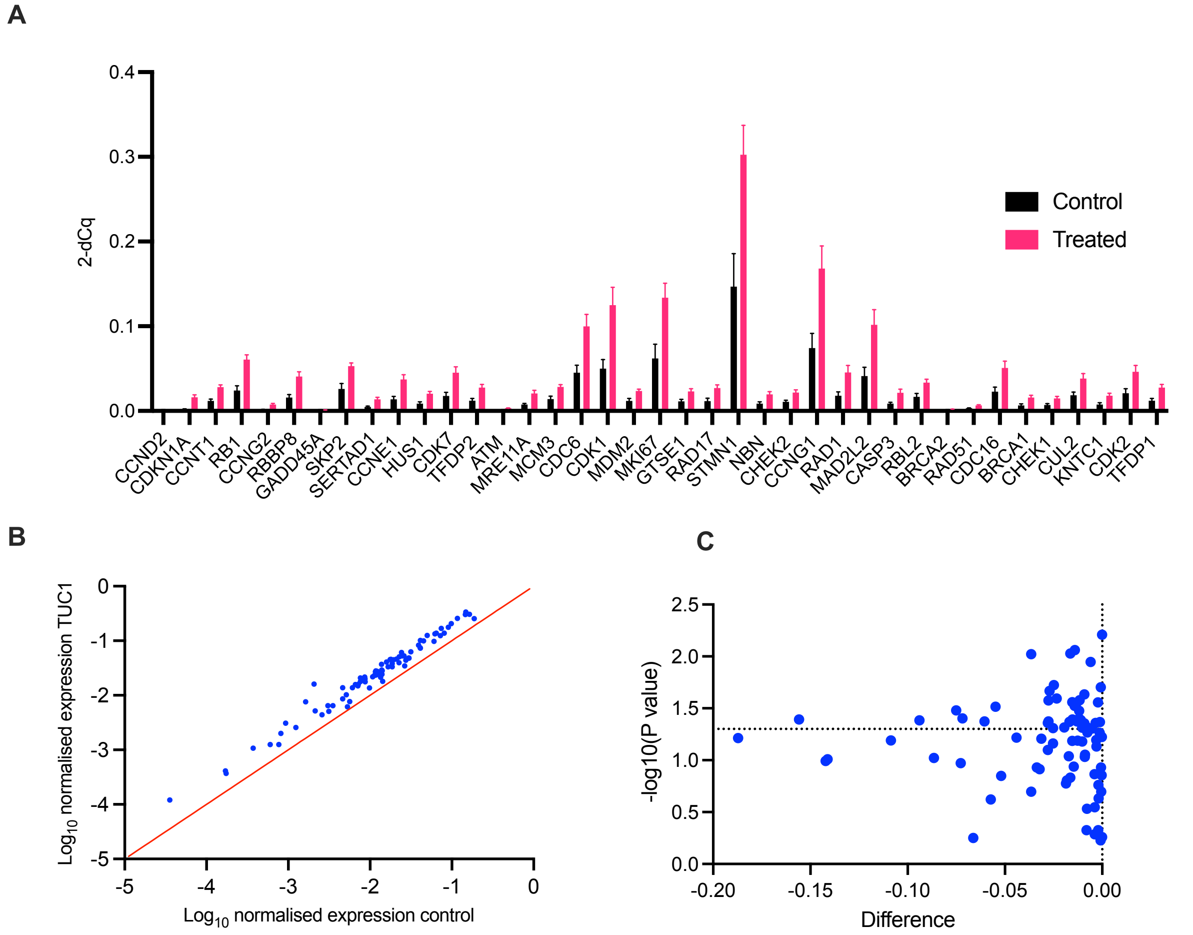

Figure S3: List of 39 genes related to DNA-repair that are statistically significantly upregulated in MIAPaCa2 cells following treatment with **1-(*S*,*R*$_P$)** (10 µM, 24 hours). A) Expression in control and treated cells expressed as 2^{-dCq} relative to *GAPDH*. B) Log normalised plot of data and C) volcano plot. The results represent the mean of three independent biological experiments (± SD, n=3).
