# Supplementary material for: A ferrocene-containing nucleoside analogue targets DNA replication in pancreatic cancer cells
Source: Metallomics. 2022 Jun 11;14(7):mfac041. doi: 10.1093/mtomcs/mfac041 (PMC9320222; doi:10.1093/mtomcs/mfac041)
Supplement: mfac041_Supplemental_Files [file mfac041_supplemental_files.zip › SupplFig4_pdf.pdf]

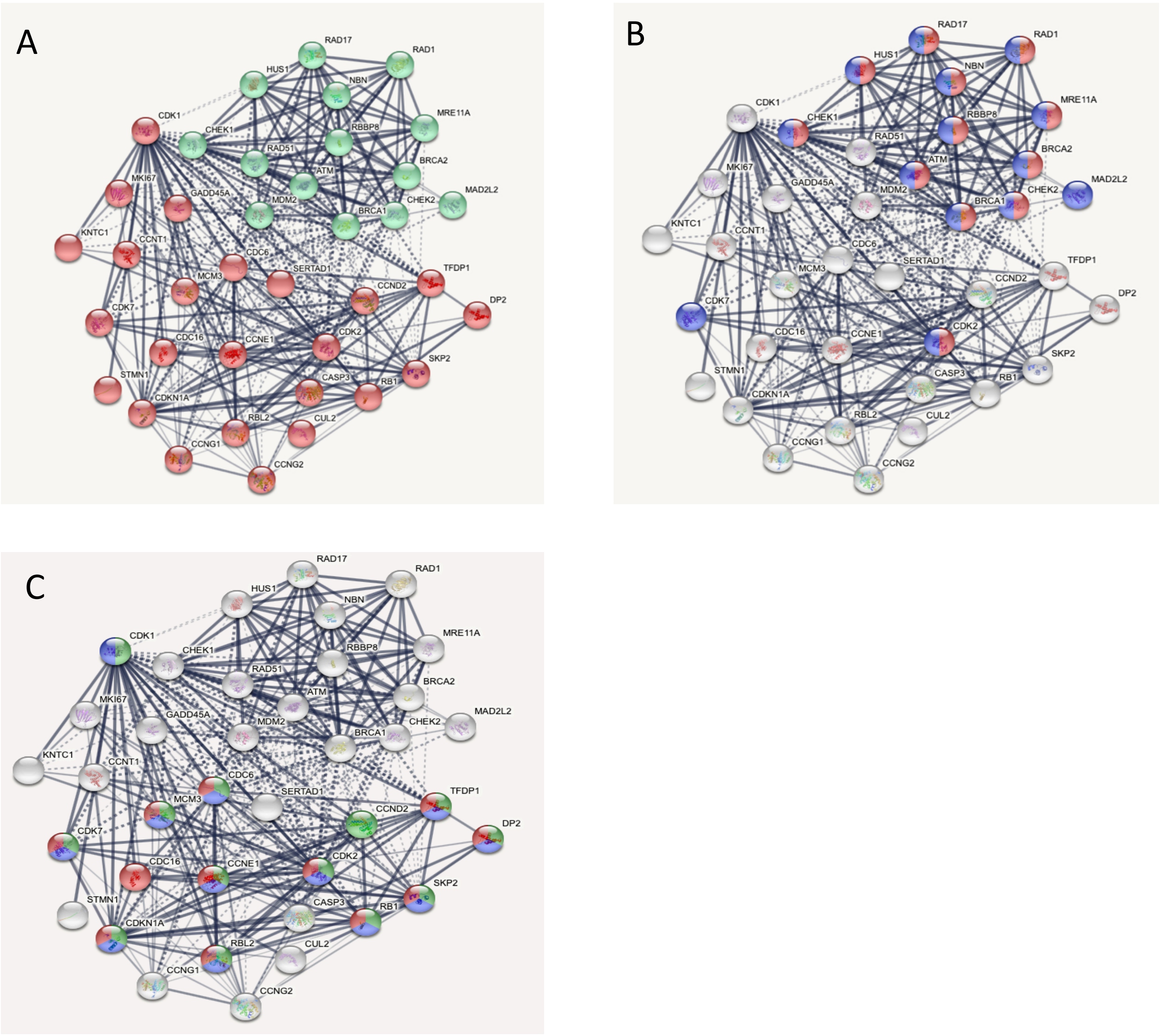

Figure S4: Kmeans clustering analysis of the 39 transcripts that were statistically significantly upregulated following treatment of MIAPaCa2 cells with **1-(*S*,*R*$_p$)** (10 μM, 24 hours). A) Overall Kmeans clustering analysis showing the separation of transcripts into 2 groups coloured red and green. B) Genes (*RAD17, RAD1, HUS1, NBN, MRE11A, CHEK1, CHEK2, RBBP8, ATM, BRCA1* and *MAD2L2*) related to DNA double strand break repair (Reactome pathway HSA-5693532 coloured red) and DNA repair (Reactome pathway HSA-73894 coloured blue) are linked to the first cluster. C) Genes (*CDK1, CDK7, MCM3, CDC6, CDC16, CCNE1, CCND2, TFDP1, DP2, SKP2, RB1, CDK2, RBL2* and *CDKN1A*) related to G1/S transition (Reactome pathway HSA-69206 coloured blue), S-phase (Reactome pathway HSA-69242 coloured green) and Mitotic G1-G1/S phase ((Reactome pathway HSA-453279 coloured red) are linked to cluster 2.
