# Supplementary material for: A ferrocene-containing nucleoside analogue targets DNA replication in pancreatic cancer cells
Source: Metallomics. 2022 Jun 11;14(7):mfac041. doi: 10.1093/mtomcs/mfac041 (PMC9320222; doi:10.1093/mtomcs/mfac041)
Supplement: mfac041_Supplemental_Files [file mfac041_supplemental_files.zip › SupplFig5_pdf.pdf]

A

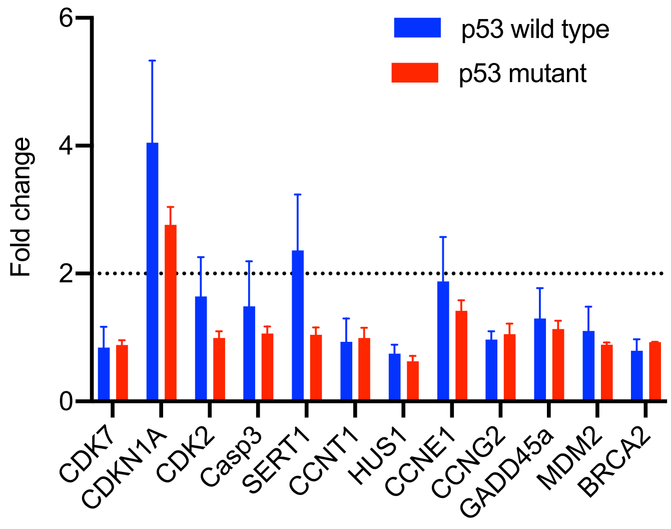

B

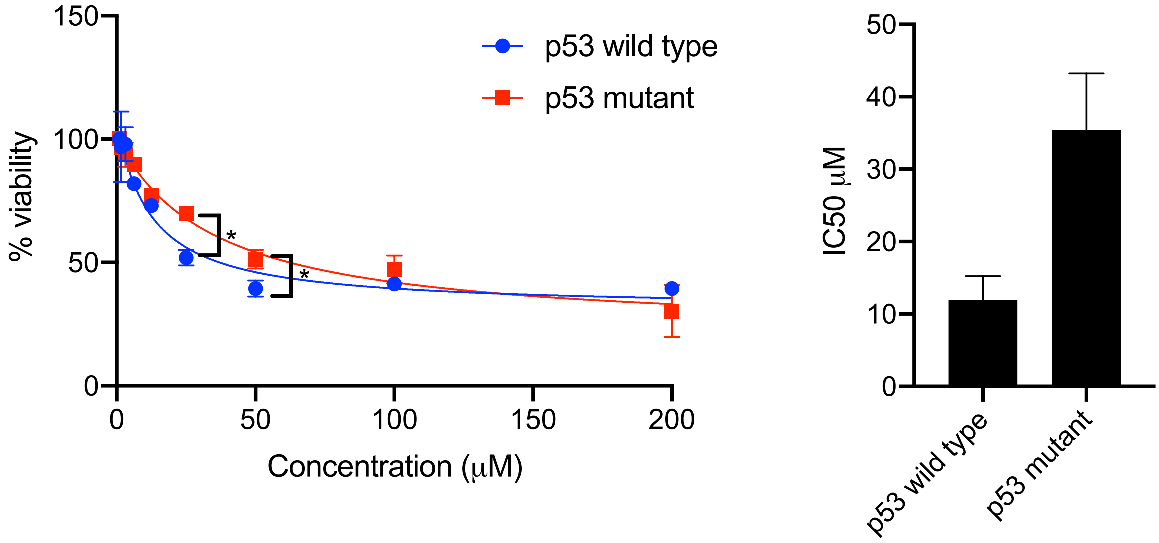

Figure S5: Transcriptional response to **1-(*S*,*R*$_p$)** is not affected by p53 status but p53 null cell lines are more sensitive to **1-(*S*,*R*$_p$)** as assessed by the MTT assay. A) Fold change in 12 genes identified in PDAC cell lines in HCT116 p53$^{+/+}$ and p53$^{-/-}$ HCT116 cells B) Cytotoxicity curves for in HCT116 p53$^{+/+}$ and p53$^{-/-}$ HCT116 cells with **1-(*S*,*R*$_p$)** for 72 hours as assessed by the MTT assay. The results represent the mean of three independent biological experiments (n=3). * There was a statistically significant difference ($P < 0.05$) in the concentration-response of wild type and mutant cells as assessed by a 2-way ANOVA followed by a *post-hoc* Tukey t-test.
