# Supplementary material for: A ferrocene-containing nucleoside analogue targets DNA replication in pancreatic cancer cells
Source: Metallomics. 2022 Jun 11;14(7):mfac041. doi: 10.1093/mtomcs/mfac041 (PMC9320222; doi:10.1093/mtomcs/mfac041)
Supplement: mfac041_Supplemental_Files [file mfac041_supplemental_files.zip › SupplFig6_pdf.pdf]

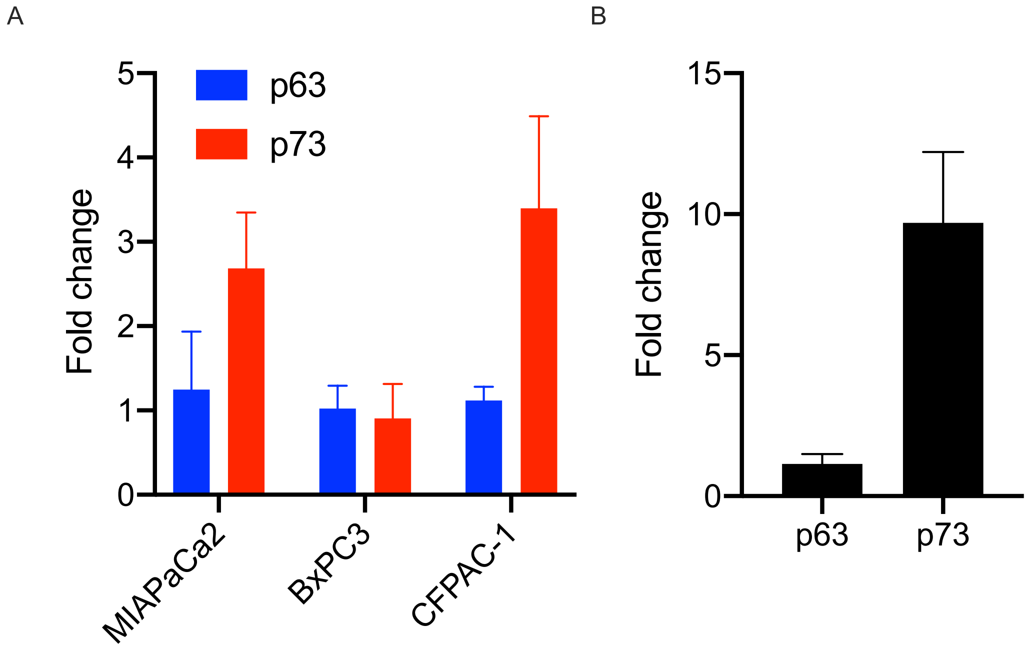

Figure S6: A) The p53 homologue p73 but not p63 is differentially regulated in PDAC cells following treatment with **1-(*S*,*R*$_p$)** as assessed by qPCR. B) p73 but not p63 is upregulated in HCT116 p53 knock cells. The results represent the mean of three independent biological experiments (n=3).
