# Supplementary material for: A ferrocene-containing nucleoside analogue targets DNA replication in pancreatic cancer cells
Source: Metallomics. 2022 Jun 11;14(7):mfac041. doi: 10.1093/mtomcs/mfac041 (PMC9320222; doi:10.1093/mtomcs/mfac041)
Supplement: mfac041_Supplemental_Files [file mfac041_supplemental_files.zip › SupplFig7_pdf.pdf]

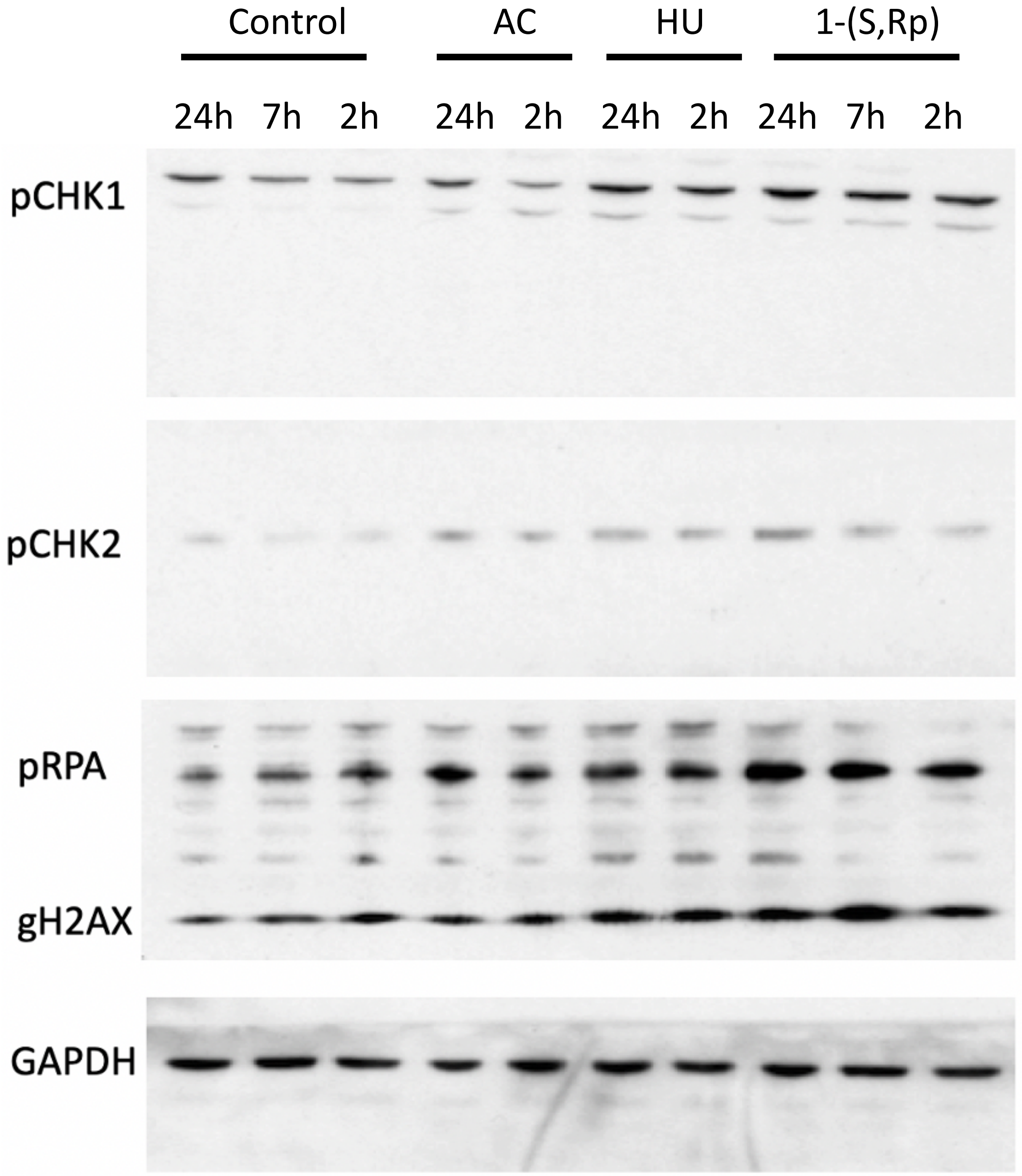

Figure S7: Western blotting analysis of cell cycle and DNA-damage response proteins in MIAPaCa2 cells. Cells were treated with 10 μM **1-(*S*,*R*$_p$)** for 2, 7 and 24 hours. As a positive control cell were treated with 0.5 μM hydroxyurea (HU) and 2.4 μM aphidicolin (AC) for 2 and 24 hours. Following treatment, cells were harvested and lysed in radioimmunoprecipitation assay (RIPA) buffer mixed with phosphatase inhibitor cocktail and protein kinase inhibitor. Lysates were centrifuged at 10 000 rpm for 5 min at 4°C. Protein quantification was carried out by Bradford assay and mixed with Laemmli buffer and 20 μg total protein loaded onto each gel. After separation on NuPAGE 4-12% Bis-Tris Gel (ThermoFisher Scientific, USA) (Cat. No. NP0322BOX), the proteins were transferred onto 0.2 μm nitrocellulose membrane that was blocked in immobilon and incubated with the primary and secondary antibodies. For detection. HRP was visualised using Luminata reagent (MerckMillipore, WBLUR0100) onto Amersham Hyperfilm ECL (GE Healthcare Life Sciences, 28906836). Antibodies used were: pChk1 (Ser 345) (1:1000) Cell Signalling Cat#2341, pChk2 (Thr 68) (1:1000) Cell Signalling Cat#2661, g-histone H2AX (Ser139)(1:250) Cell Signalling Cat# 9718, pRPA (RPA2) (Ser4/Ser8), GAPDH (1:1000) Genetex Cat#GTX627408. Secondary antibodies used were goat anti-mouse HRP conjugated (Fisher Scientific, PA1-74421) and goat anti-rabbit HRP conjugated (Fisher Scientific, PI-31460). A representative gel from two independent repeats is shown.
