# Supplementary material for: A ferrocene-containing nucleoside analogue targets DNA replication in pancreatic cancer cells
Source: Metallomics. 2022 Jun 11;14(7):mfac041. doi: 10.1093/mtomcs/mfac041 (PMC9320222; doi:10.1093/mtomcs/mfac041)
Supplement: mfac041_Supplemental_Files [file mfac041_supplemental_files.zip › Supplementary_Methods_pdf.pdf]

Supplementary Materials and Methods

p53 genotyping of PDAC cell lines by sequencing

MIAPaCa2, BxPC3 and CFPAC-1 cells were seeded at a density of 3 x 10^5 cells/mL and incubated in 5% CO_2, 37°C overnight. RNA was extracted using the RNeasy Mini Kit (Qiagen, Hilden, Germany) following manufacturers guidelines. This was followed by cDNA synthesis using Tetro CDNA synthesis kit (Bioline, London, UK). A region of ~175 bp covering mutations for all three cell lines was amplified using Phusion High-Fidelity PCR Kit (ThermoFisher Scientific, USA) (Cat. no. F553S). Sequences for the primers used to amplify the region are shown below. PCR products were resolved on a 1% agarose gel in TAE buffer followed by gel purification using QIAquick Gel Extraction Kit (Qiagen, Germany) (Cat. no. 28704). Samples were submitted for sequencing by the Genomics Facility at the University of Birmingham and analysed using SnapGene (https://www.snapgene.com/snapgene-viewer/).

	Forward Primer	Reverse Primer
Set 1	5-tcgacatagtgtggtggtgc	5-aaagctgttccgtcccagta
Set 2	5-acttttcgacatagtgtggtgg	5-tcaaagctgttccgtccca

Confirmation of p53 genotype in:

A) MIAPaCa2, (pR248W, c742 C→T)

Homo sapiens tumor protein p53 (TP53), transcript variant 1, mRNA

Sequence ID: NM_000546.6 Length: 2512 Number of Matches: 1

Range 1: 814 to 952 GenBank Graphics ▼ Next Match ▲ Previous

Score	Expect	Identities	Gaps	Strand
252 bits(136)	4e-63	138/139(99%)	0/139(0%)	Plus/Plus

```
Query  23   GGTTGGCTCTGACTGTACCACCATCCACTACAACTACATGTGTAACAGTTCCTGCATGGG  82
            ||||||||||||||||||||||||||||||||||||||||||||||||||||||||||||
Sbjct  814  GGTTGGCTCTGACTGTACCACCATCCACTACAACTACATGTGTAACAGTTCCTGCATGGG  873

Query  83   CGGCATGAACTGGAGGCCCATCCTCACCATCATCACACTGGAAGACTCCAGTGGTAATCT  142
            |||||||||| |||||||||||||||||||||||||||||||||||||||||||||||||
Sbjct  874  CGGCATGAACCGGAGGCCCATCCTCACCATCATCACACTGGAAGACTCCAGTGGTAATCT  933

Query  143  ACTGGGACGGAACAGCTTT  161
            |||||||||||||||||||
Sbjct  934  ACTGGGACGGAACAGCTTT  952
```

B) BxPC3, (pY220C, c659 A→G)

Range 1: 773 to 908 GenBank Graphics ▼ Next Match ▲ Previous

Score	Expect	Identities	Gaps	Strand
233 bits(126)	1e-57	133/136(98%)	2/136(1%)	Plus/Plus

```
Query  3    ACTTTTCGACATAGTGTGGTGGTGCCCTGTGAGCCGCCTGAGGTTGGCTCTGACTGTACC  62
            |||||||||||||||||||||||||||| |||||||||||||||||||||||||||||||
Sbjct  773  ACTTTTCGACATAGTGTGGTGGTGCCCTATGAGCCGCCTGAGGTTGGCTCTGACTGTACC  832
```

C) CFPAC-1, (pC242R, c724 T→C)

Sequence of primers used for qPCR quantification of the 13 test and 2 housekeeping genes

Gene	Forward Primer	Reverse Primer
BRCA2	GTTTCCACACCTGTCTCAGC	GGTGGAGGTAAAGGCAGTCT
SERTAD1	GCCGTTTCCTGATTGGTTGT	AGACCCTTGCTCAGCATCTT
HUS1	GACTTGGTGTAGTAGCCAGAA	CGGGGTGAACACACTGAAAT
GADD45A	CACTGTCGGGGTGTACGAAG	CCTGGATCAGGGTGAAGTGG
Casp3	GCCTCTTCCCCCATTCTCAT	CTTCCATGTATGATCTTTGGTTCC
MDM2	CGAGCTTGGCTGCTTCTG	GTACGCACTAATCCGGGGAG
CDKN1A	GCCGAAGTCAGTTCCTTGTG	CATGGGTTCTGACGGACATC
CDK2	CATCTTTGCTGAGATGGTGACT	ACTTGGCTTGTAATCAGGCAT
CCNE1	CCCATCATGCCGAGGGAG	CACGTTTGCCTTCCTCTTCC
CDK7	CTCGGGCAAAGCGTTATGAG	CTCTGGCCTTGTAAACGGTG
CCNT1	TGGAAAATAGCCCATCCCGT	GTGAGACGTTAAGACGCTGC
CCNG2	AGGTGAGGCTACAGTGATTCC	AGGCACAGATGCCAAACCTA
B2M	TTTGGCTCACAGTGTAAAGGG	GTCACCCCAACTATGCCATT
RBBP8	CTCAGAAAGTGCTCGCTTCC	TCTGCAGAGTTAGGGCTTCC
TBP	CCGGCTGTTTAACTTCGCTT	CACACGCCAAGAAACAGTGA

References

[1] M.M. Bradford, A rapid and sensitive method for the quantitation of microgram quantities of protein utilizing the principle of protein-dye binding, Anal. Biochem., 72 (1976) 248-254.
